# USP16-mediated histone H2A lysine-119 deubiquitination during oocyte maturation is a prerequisite for zygotic genome activation

**DOI:** 10.1093/nar/gkac468

**Published:** 2022-05-30

**Authors:** Yan Rong, Ye-Zhang Zhu, Jia-li Yu, Yun-Wen Wu, Shu-Yan Ji, Yong Zhou, Yu Jiang, Jin Jin, Heng-Yu Fan, Li Shen, Qian-Qian Sha

**Affiliations:** Key Laboratory of Reproductive Dysfunction Management of Zhejiang Province; Assisted Reproduction Unit, Department of Obstetrics and Gynecology, Sir Run Run Shaw Hospital, School of Medicine, Zhejiang University, Hangzhou 310016, China; MOE Key Laboratory for Biosystems Homeostasis & Protection and Innovation Center for Cell Signaling Network, Life Sciences Institute, Zhejiang University, Hangzhou 310058, China; MOE Key Laboratory for Biosystems Homeostasis & Protection and Innovation Center for Cell Signaling Network, Life Sciences Institute, Zhejiang University, Hangzhou 310058, China; MOE Key Laboratory for Biosystems Homeostasis & Protection and Innovation Center for Cell Signaling Network, Life Sciences Institute, Zhejiang University, Hangzhou 310058, China; MOE Key Laboratory for Biosystems Homeostasis & Protection and Innovation Center for Cell Signaling Network, Life Sciences Institute, Zhejiang University, Hangzhou 310058, China; MOE Key Laboratory for Biosystems Homeostasis & Protection and Innovation Center for Cell Signaling Network, Life Sciences Institute, Zhejiang University, Hangzhou 310058, China; Fertility Preservation Laboratory, Reproductive Medicine Center, Guangdong Second Provincial General Hospital, Guangzhou 510317, China; MOE Key Laboratory for Biosystems Homeostasis & Protection and Innovation Center for Cell Signaling Network, Life Sciences Institute, Zhejiang University, Hangzhou 310058, China; MOE Key Laboratory for Biosystems Homeostasis & Protection and Innovation Center for Cell Signaling Network, Life Sciences Institute, Zhejiang University, Hangzhou 310058, China; MOE Key Laboratory for Biosystems Homeostasis & Protection and Innovation Center for Cell Signaling Network, Life Sciences Institute, Zhejiang University, Hangzhou 310058, China; MOE Key Laboratory for Biosystems Homeostasis & Protection and Innovation Center for Cell Signaling Network, Life Sciences Institute, Zhejiang University, Hangzhou 310058, China; Fertility Preservation Laboratory, Reproductive Medicine Center, Guangdong Second Provincial General Hospital, Guangzhou 510317, China

## Abstract

Maternal-to-zygotic transition (MZT) is the first and key step in the control of animal development and intimately related to changes in chromatin structure and histone modifications. H2AK119ub1, an important epigenetic modification in regulating chromatin configuration and function, is primarily catalyzed by PRC1 and contributes to resistance to transcriptional reprogramming in mouse embryos. In this study, the genome-wide dynamic distribution of H2AK119ub1 during MZT in mice was investigated using chromosome immunoprecipitation and sequencing. The results indicated that H2AK119ub1 accumulated in fully grown oocytes and was enriched at the TSSs of maternal genes, but was promptly declined after meiotic resumption at genome-wide including the TSSs of early zygotic genes, by a previously unidentified mechanism. Genetic evidences indicated that ubiquitin-specific peptidase 16 (USP16) is the major deubiquitinase for H2AK119ub1 in mouse oocytes. Conditional knockout of *Usp16* in oocytes did not impair their survival, growth, or meiotic maturation. However, oocytes lacking USP16 have defects when undergoing zygotic genome activation or gaining developmental competence after fertilization, potentially associated with high levels of maternal H2AK119ub1 deposition on the zygotic genomes. Taken together, H2AK119ub1 level is declined during oocyte maturation by an USP16-dependent mechanism, which ensures zygotic genome reprogramming and transcriptional activation of essential early zygotic genes.

## INTRODUCTION

Gametes are highly differentiated cell types. During oogenesis, oocytes gain competence in accomplishing meiotic maturation and prepare for embryonic development following fertilization (1). Fertilization of the oocyte by sperm requires major epigenetic remodeling to reconcile the two parental genomes and allow for the formation of a totipotent zygote (2). In particular, the paternal genome arrives densely packed with protamines rather than histones, and the maternal epigenome is highly specialized (3). During the maternal-to-zygotic transition (MZT), parental genomes undergo drastic reprogramming (4). The dynamic histone posttranslational modifications, including acetylation, phosphorylation, methylation and ubiquitination, mediate a wide range of nuclear events during these processes (5–9). Specific maternal factors must unravel these specialized chromatin states to enable zygote genome activation (ZGA) and development to proceed.

The incorrect settings of these histone markers in cloned animals have been correlated with their poor development potential. Using nuclear transfer to *Xenopus* and mouse oocytes, DNA methylation, H2AK119 ubiquitination, and H3K9 and H3K27 trimethylation have been identified as major epigenetic configurations that directly resist transcriptional reprogramming by oocyte factors (10–13). However, the regulators underlying these dynamic histone modifications post fertilization and their impact on the zygotic genome reprogramming remain to be further explored.

Monoubiquitination of histone H2A at lysine-119 (H2AK119ub1) by polycomb repressive complex 1 (PRC1) is a non-proteolytic modification (14). H2AK119ub1 alters the chromatin structure and plays a role in the developmental regulation of various multicellular organisms (15). PRC1 is composed of E3 ubiquitin ligase RING1, its homolog RNF2, and their regulatory subunits. PRC1 acts in the oocytes to establish developmental competence after fertilization by silencing differentiation-inducing genes and defining appropriate chromatin states (16). Genetic ablation of *Ring1* and *Rnf2* in oocytes results in loss of H2AK119ub1 accumulation, induction of massive transcriptional misregulation during oocyte growth and a developmental arrest at the two-cell stage of embryogenesis (17). However, the H2AK119ub1 on chromosomes is nearly undetectable in wild-type (WT) MII oocytes (17), arguing that this modification may not be passed on directly from mammalian gametes to the next generation.

The level and distribution of H2AK119ub1 on chromatin are highly dynamic, as has been shown in mouse early embryos during preimplantation development (18). H2AK119ub1 signal is already present in both male and female pronuclei immediately after fertilization, but the H2AK119ub1 level fluctuates during pronuclear development and chromatin reorganization. H2AK119ub1 is also detected in the nuclei of blastomeres of preimplantation embryos; however, its intranuclear distribution shows developmental stage-associated changes. By developing highly sensitive chromatin immunoprecipitation sequencing (ChIP-seq) approaches, several groups have described the landscape of histone H3 modifications, including K4, K9, K27 and K36 trimethylations, from the developing gamete stage to the post-implantation embryo stage (8,19–22). These epigenetic markers have both canonical and non-canonical patterns of distribution in gametes, undergo drastic redistributions during MZT, and are associated with genome reprogramming. Latest reports have talked about the genome-wide distribution of H2AK119ub1 in mammalian oocytes and early embryos (23,24). Loss of H2AK119ub1, but not H3K27me3, in zygotes leads to premature activation of developmental genes during ZGA and subsequent embryonic arrest (23). PRC1 was reported to play key roles in the establishment of H2AK119ub1 marks of oocytes and early embryos (17,24), even so, enzymes that antagonize with PRC1 and remove H2AK119ub1 from chromatin during oocyte maturation and maternal-to-zygotic transition have not been investigated.

H2AK119ub1 can be deubiquitinated by a group of deubiquitinases (DUBs), also called ubiquitin-specific peptidases (USPs), currently known to include USP3, 12, 16, 21, 22, 28, 32 and 36, BAP1 and MYSM1 (25–32). Among them, USP16 (also called Ubp-M) was the first DUB of H2A to be identified and absence of USP16 resulted in an increase of H2AK119ub1 (not H2BK120ub1) level (14,27). RNAi depletion of *Usp16* in HeLa cells results in slow cell growth rates owing to impaired mitosis (27). *Usp16* knockout results in a remarkable increase of H2AK119ub1 level and the inhibition of lineage-specific gene expression in ESCs, followed by embryonic lethality in mice (14). Conditional knockout of *Usp16* in the bone marrow results in a significant increase in the global H2AK119ub1 level and a dramatic reduction in mature and progenitor cell populations (33). *Usp16* has received particular attention in recent years because its location on chromosome 21 and is a key epigenetic switch that regulates stem cell self-renewal and senescence in Down syndrome (34,35). As a deubiquitinase, USP16 also regulates the mono-ubiquination of other substrates. For example, USP16 counteracts mono-ubiquitination of RPS27a and promotes maturation of the 40S ribosomal subunit (36), and USP16 regulates the deubiquitination of calcineurin A to control peripheral T cell maintenance (37). However, the function of USP16 in germ cells is unclear. It remains undetermined if USP16 is the major H2AK119 DUB in oocytes and if it plays an indispensable function in the events of meiosis and genome reprogramming during MZT.

In this study, we investigated the dynamic distribution of H2AK119ub1 and role of USP16 in mouse oogenesis and MZT. H2AK119ub1 accumulates during oogenesis but was promptly declined from the condensing chromosomes during oocyte meiotic maturation. ChIP-seq studies revealed that H2AK119ub1 bound to the transcription start sites (TSSs) of many important genes that regulate early embryogenesis. We discovered that conditional deletion of *Usp16* in oocytes as early as at the primordial follicle stage did not impair ovarian follicle development or oocyte meiosis, but blocked the H2AK119ub1 removal prior to MZT. As a result, *Usp16* deletion prevented ZGA at the two-cell stage and reduced the developmental potential of preimplantation embryos.

## MATERIALS AND METHODS

### Mice

*Usp16^fl/fl^;Gdf9-Cre* mice were produced by crossing the mice bearing the *Usp16^fl^* allele with *Gdf9-Cre* transgenic mice (38). The insertion sites of LoxP have been illustrated in Supplementary Figure S4A. All WT (*Usp16^+/+^*) and cKO (*Usp16^fl/fl^;Gdf9-Cre*) mice had a C57BL/6 genetic background. Mice were maintained under SPF conditions in a controlled environment of 20–22°C, with a 12/12 h light/dark cycle, 50–70% humidity, and food and water provided *ad libitum*. Experimental procedures and animal care were in accordance with the Animal Research Committee guidelines of Zhejiang University.

### Fertility evaluation

Two-month-old WT and *Usp16^fl/fl^;Gdf9-Cre* females (n = 5 for each genotype) were maintained in the same cage with a fertile WT male for at least 6 months. Delivery frequency and litter size were documented on day 1 of the females giving birth.

### Oocyte collection and culture

Mice (21–23 days old) were injected with 5 IU of PMSG and humanely euthanized 44 h later. Oocytes at the GV stage were harvested in M2 medium (M7167; Sigma-Aldrich) and cultured in mini-drops of M16 medium (M7292; Sigma-Aldrich) covered with mineral oil (M5310; Sigma-Aldrich) at 37°C in a 5% CO_2_ atmosphere. In some experiments, milrinone (2 μM) was added to the culture media to inhibit spontaneous GVBD.

### Superovulation and fertilization

Female mice (21–23 days old) were intraperitoneally injected with 5 IU of PMSG followed by hCG 44 h later. Oocyte and cumulus complexes were harvested from the oviducts 16 h after hCG injection. The numbers of oocytes were counted after digestion with 0.3% hyaluronidase. To obtain early embryos, superovulated female mice were mated with 10–12-week-old WT males. Successful mating was confirmed by the presence of vaginal plugs. Zygotes were harvested from the oviducts 28 h after hCG injection.

### Immunofluorescence

Oocytes were fixed in 4% paraformaldehyde in PBS for 30 min and permeabilized in PBS containing 0.3% Triton X-100 for 20 min. After being blocked with 1% bovine serum albumin in PBS, the oocytes were incubated with primary antibodies for 1 h and sequentially labeled with Alexa Fluor 594- or 488-conjugated secondary antibodies (Molecular Probes) and 4′,6-diamidino-2-phenylindole (DAPI) for 30 min. Imaging of oocytes after immunofluorescence was performed on a LSM710 confocal microscope (Zeiss). The antibodies used are listed in Supplementary Table S1.

### Chromosome spreading and immunofluorescence

Zona pellucida-free oocytes were fixed in a solution containing 1% paraformaldehyde, 0.15% Triton X-100 and 3 mmol/L DTT on glass slides for 30 min and air-dried. Immunofluorescent staining was performed as in oocytes described above.

### Plasmid construction, *in vitro* mRNA synthesis and microinjection

The cDNA encoding human USP16 (NP_001001992.1) was PCR amplified and cloned into a pcDNA3-derived expression vector; it was then fused with an N-terminal mCherry tag. The USP16^C205S^ mutation was generated by PCR-based point mutagenesis. Plasmids were liberalized using appropriate restriction enzymes. Then, 5′-capped mRNAs were synthesized using Sp6 or T7 mMESSAGE mMACHINE Kit (Invitrogen, AM1340 or AM1344) and poly (A) tails were added using a Poly (A) Tailing Kit (Invitrogen, AM1350). The synthesized mRNA was recovered using lithium chloride precipitation and dissolved in nuclease-free water.

For microinjection, GV oocytes were collected in M2 medium supplemented with 2 μM milrinone to inhibit spontaneous germinal vesicle breakdown. All microinjections were performed using an Eppendorf Transferman NK2 micromanipulator. Approximately 10 pl synthetic mRNAs (500 μg/ml) were microinjected into the ooplasm. After injection, the oocytes were cultured at 37°C with 5% CO_2_.

### Detection of transcription and protein synthesis in oocytes/embryos

To detect transcription activity, the oocytes/embryos were cultured in M16 medium containing 1 mM 5-ethynyl uridine (EU) for 1 h. EU staining was performed using Click-iT^®^ RNA Alexa Fluor^®^ 488 Imaging Kit (Life Technologies), according to the manufacturer's instructions.

To detect protein synthesis, oocytes/embryos were cultured in M16 medium supplemented with 50 μM l-homopropargylglycine (HPG) for 1 h. HPG was detected using Click-iT^®^ HPG Alexa Fluor^®^ Protein Synthesis Assay Kit (Life Technologies), according to the manufacturer's instructions. The mean cytoplasmic signal was measured across the middle of each oocyte/embryo and quantified using ImageJ software.

### ChIP-seq library preparation and sequencing

ChIP-seq was performed following the previously reported carrier DNA assisted ChIP-seq (CATCH-Seq) protocol (39,40). For each immunoprecipitation assay, 200–500 oocytes, zygotes or two-cell embryos, as well as one microgram of H2AK119ub1 antibody (8240, Cell Signaling Technology) was used. The precipitated DNA was processed into sequencing libraries using the NEBNext Ultra II DNA Library Prep Kit for Illumina (E7645, New England Biolabs) following the manufacturer's instructions. Barcoded libraries were pooled and sequenced on the Illumina HiSeq X Ten platform.

### ChIP-seq data analysis

ChIP-seq reads were trimmed to 50 bp and aligned to the mouse genome (build mm9) using bowtie2 (v2.3.4.1) with default parameters. All unmapped reads, non-uniquely mapped reads and PCR duplicates were removed. H3K4me3 peaks were called using MACS2 (v 2.1.1.20160309) with the following parameters ‘-q 0.05 –nomodel –nolambda –broad –extsize 300 -B –SPMR -g mm’ and signal tracks for each sample were generated with chromstaR (v1.7.3) with parameter binsizes = 1000, stepsizes = 200. In order to compare ChIP-seq data across different stages, signal tracks were further normalized using random regions. When defining H2AK119ub1 peaks, low quality peaks (RPKM < 1, width < 800) were excluded. Heatmaps were generated using deepTools (v2.5.4). Promoters were defined as +/-2kb regions flanking annotated TSS. The H2AK19ub1 intensity of promoter regions was calculated using the bedtools (v2.26.0) ‘coverage’ tool and statistically significant differences in the H2AK119ub enrichment between various stages were identified using ‘DESeq2’ R package. H3K4me3/ H3K27me3 ChIP-seq and RNA-seq data for GV oocytes were downloaded from the GEO database (GSE71434 and GSE76687).

### RNA-seq library preparation and gene expression analysis

Samples (10 embryos per sample) were collected from WT and *Usp16^fl/fl^;Gdf9-Cre* female mice for RNA-seq. Each sample was directly lysed with 4 μl lysis buffer and immediately used for cDNA synthesis using the published Smart-seq2 method. Raw reads were trimmed to 50 bp and were mapped to the mouse genome (mm9) using Tophat v2.1.1 with default parameters. The expression level of each gene was quantified as normalized FPKM (fragments per kilobase of exon per million mapped fragments) using Cufflinks v2.2.1. To identify genes functionally vital to oocytes and to avoid dramatic fold changes due to small changes of low FPKM values, genes with FPKM < 1 in all samples were excluded, and for the remaining genes, all FPKM values smaller than 1 were set to 1 in subsequent analyses. The ZGA genes were defined as genes with FC [two-cell/zygote] > 3.

### Improved single-embryo real time PCR

This method was modified from the RNA Smart-seq protocol. In brief, 6 oocytes/embryos were lysed in 2 μl lysis buffer (0.2% Triton X-100 and 2 IU/μl RNase inhibitor) followed by hybridization with random primers (Takara) and dNTP (Takara) at 72°C for 3 min and quick chill on ice. Then the samples were treated for reverse transcription and amplification with SuperScript II reverse transcriptase (Thermo Fisher Scientific) solution system at 42°C for 90 min, ending at 70°C for 15 min to inactivate the reaction. The reverse transcription products were diluted and used as templates for RT-qPCR. Quantitative RT-PCR was performed using a Power SYBR Green PCR Master Mix (Applied Biosystems, Life Technologies) on the ABI 7500 Real-Time PCR system (Applied Biosystems) with primers listed in Supplementary Table S2. Relative mRNA levels were calculated by normalizing to the levels of endogenous *Gapdh* mRNA (used as an internal control).

### Absolute RT-qPCR

The coding sequence fragments of *Usp16/21* were PCR amplified from the mouse oocyte cDNA pool wcith their respective PCR primers. Then the *Usp16/21* PCR products were inserted into the plasmid containing *Gfp* cDNA, respectively. RT-qPCR was performed using *Usp16/21* and *Gfp* primers and the two plasmids as templates. Respective cycle thresholds (Cts) for *Usp16/21* and *Gfp* were determined using ABI 7500 Real-Time PCR system (Applied Biosystems). Relative *Usp16* and *Usp21* DNA fragments levels were calculated by normalizing to the levels of *Gfp* DNA fragments. Their respective levels were calculated by 2^–ΔCt^, so the primer efficiency of *Usp16* and *Usp21* is 2 ^–(Ct1-Ct3)^/2 ^–(Ct2-Ct4)^; Relative *Usp16* and *Usp21* mRNA levels were calculated by normalizing to the levels of endogenous *Gapdh* mRNA. Their respective levels are calculated by 2^–ΔCt (^*^Usp16 or Usp21-Gapdh^*^)^. Then, relative ratio between *Usp16* and *Usp21* mRNA is 2^–ΔCt (^*^Usp16-Gapdh^*^)^/2 ^–ΔCt (^*^Usp21-Gapdh^*^)^. Absolute ratio between *Usp16* and *Usp21* mRNA is that relative ratio divided by primer efficiency.

### Parthenogenetic activation

Oocytes were collected from oviducts 16 h after hCG injection, denuded by 0.3% hyaluronidase treatment, and cultured in Ca^2+^-free CZB buffer with 1 mmol/ml SrCl_2_·6H_2_O for 2–4 h. After 3 h, Parthenogenetic activated oocytes were washed and cultured in KSOM (Millipore) drops at 37°C with 5% CO_2_.

### Histological analysis

Ovaries were collected and then fixed in formalin overnight, processed, and embedded in paraffin using standard protocols. Ovaries were sectioned at 5 μm and stained with hematoxylin and eosin. Immunohistochemistry was performed using standard protocols. The antibodies used are listed in Supplementary Table S1.

### Western blot analysis

Oocytes were lysed in protein loading buffer and heated at 95°C for 10 min. SDS-PAGE and immunoblots were performed following standard procedures using a Mini-PROTEAN Tetra Cell System (Bio-Rad, Hercules, CA, USA). The antibodies used are listed in Supplementary Table S1.

### Statistical analysis

Results have been expressed as mean ± SEM. Most experiments included at least three independent samples and were repeated at least three times. Results for two experimental groups were compared using two-tailed unpaired Student's *t*-tests. Statistically significant values of *P*< 0.05, *P*< 0.01, and *P*< 0.001 by two-tailed Student's *t*-test have been indicated using asterisks (*), (**), and (***), respectively. ‘n.s.’ indicates non-significant.

## RESULTS

### Mono-ubiquitination of histone H2A at lysine-119 is dynamic in oocytes and early embryos

**Figure 1.**
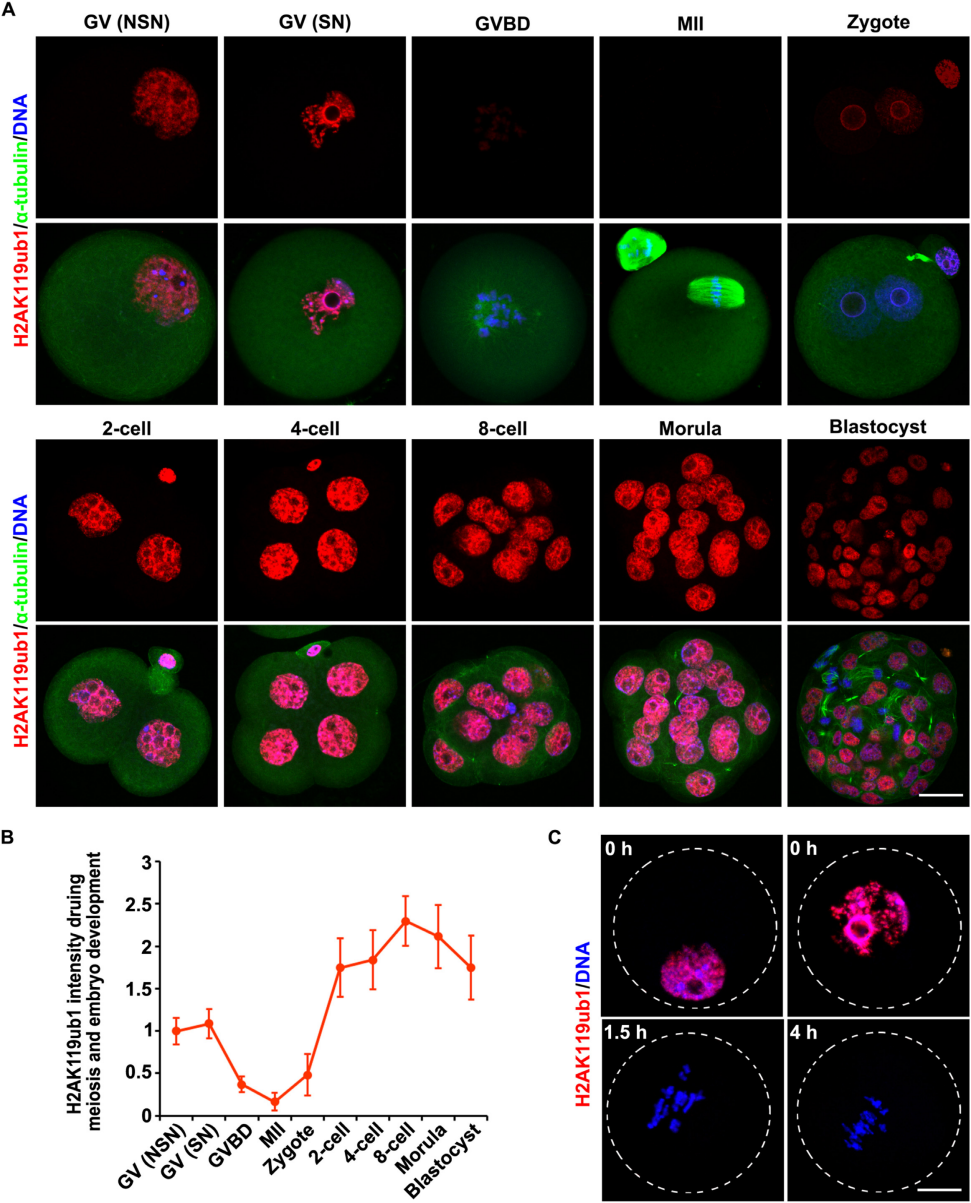
Dynamic changes in H2AK119ub1 levels in oocytes and early embryos. (**A** and **C**) H2AK119ub1 immunofluorescence results in mouse oocytes and preimplantation embryos at different developmental stages. NSN, non-surrounded nucleolus; SN, surrounded nucleolus. Scale bar, 20 μm. (**B**) Quantification of H2AK119ub1 signal intensity of (A). The numbers of analyzed oocytes or embryos at each stage were more than eight.

In WT oocytes, including the non-surrounded nucleolus (NSN) and the surrounded nucleolus (SN) types (41), chromosomal H2AK119ub1 was found to be at a high level (Figure 1A-B). After germinal vesicle breakdown (GVBD), however, the H2AK119ub1 level significantly decreased and was almost undetectable during meiotic maturation. After fertilization, H2AK119ub1 started to re-accumulate in both pronuclei. The H2AK119ub1 level then increased during development from the two-cell stage to the morula stage and decreased a little at the blastocyst stage (Figure 1A-B), as previously reported (18). Immunofluorescence results showed that H2AK119ub1 was rapidly declined from the chromatin within 1.5 h after release from germinal vesicle (GV) stage-arrest of the oocytes (Figure 1C), suggesting that the decrease of H2AK119ub1 level might be coupled with nuclear envelope breakdown. Unfortunately, we could not detect endogenous H2AK119ub1 by western blot in GV oocytes using the commercially available antibodies, either because the antibodies were not sensitive enough or because H2AK119ub1 was not abundant in the oocytes.

In addition, we noticed that H2AK119ub1 also diminished during M-phase in dividing blastomeres of the blastocyst (Figure 1A). Did H2AK119ub1 diminish in each division phase? We collected more embryos at different developmental stages and found that chromosomal H2AK119ub1 labeling was still present in mitotic blastomeres of the 2-, 4- and 8-cell stage embryos but this labeling disappeared during mitosis of morula and in blastocyst stage embryos, as that in meiosis (Supplementary Figure S1A, B). We speculated that the diminishment of H2AK119ub1 on mitotic chromosomes in blastocysts might be related to cell lineage specifications rather than to the maternal-to-zygotic transition. Therefore, the decrease of H2AK119ub1 levels during meiotic division was unique and meaningful.

### Deubiquitinase USP16 is highly expressed in mammalian oocytes and early embryos

**Figure 2.**
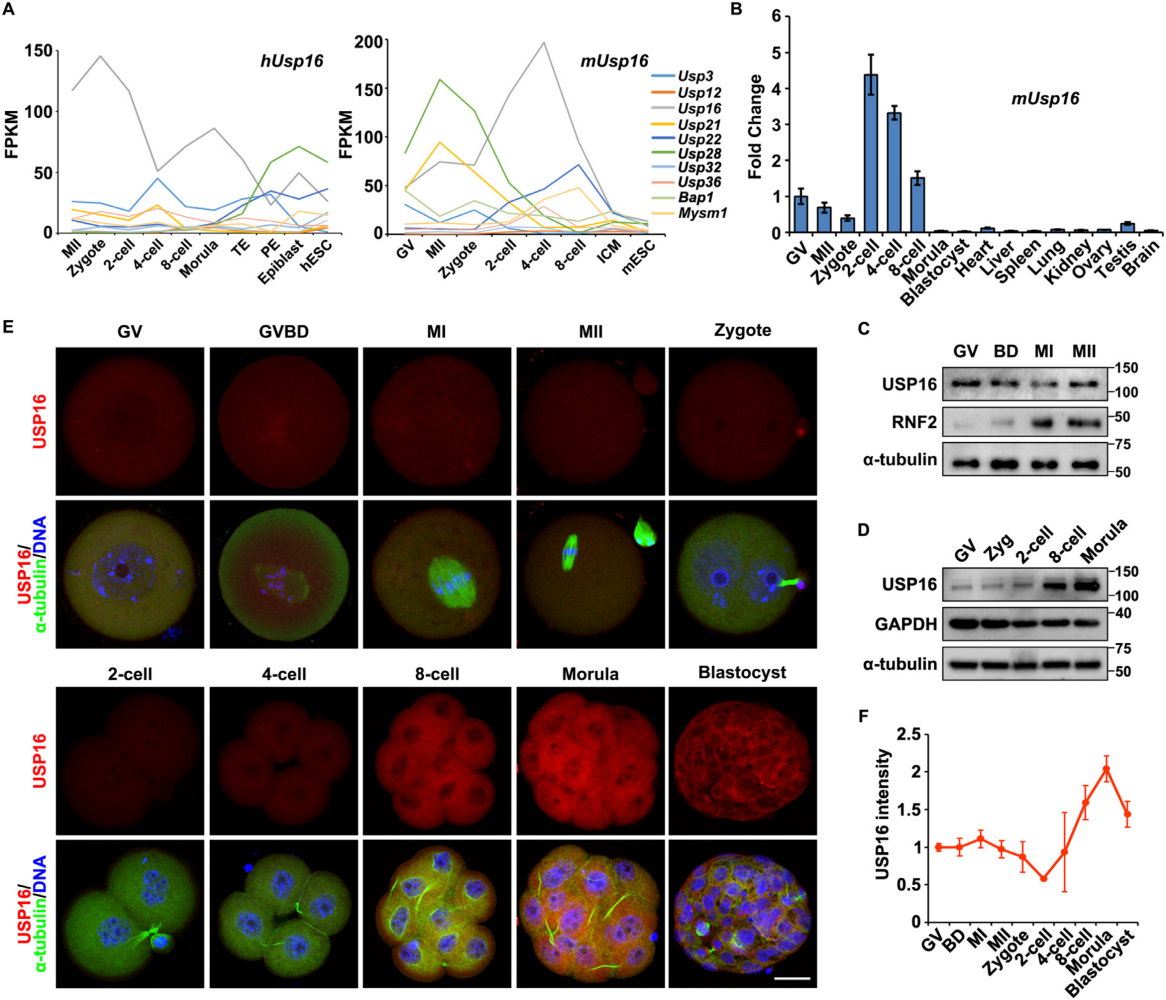
Expression of potential H2AK119 deubiquitinating enzymes in oocytes and early embryos. (**A**) mRNA profiles of putative H2A deubiquitinases detected in human (left) and mouse (right) oocytes and early embryos using RNA-seq (20,42). TE, trophectoderm; PE, primitive endoderm; ICM, inner cell mass. (**B**) RT-qPCR results showing relative expression levels of mouse *Usp16* in oocytes, preimplantation embryos and somatic tissues. *Gapdh* was used as an internal control. *n* = 3 biological replicates. (**C**) Western blot results showing USP16 and RNF2 expression levels in oocytes during meiotic maturation. Total proteins from 100 oocytes were loaded in each lane. α-tubulin was blotted as a loading control. (**D**) Western blot results showing USP16 expression levels in embryos at different developmental stages. Total proteins from 100 oocytes were loaded in each lane. GAPDH and α-tubulin was blotted as a loading control. (**E**) USP16 immunofluorescence results in mouse oocytes and preimplantation embryos. Scale bar, 20 μm. (**F**) Quantification of USP16 signal intensity of (E). The numbers of analyzed oocytes or embryos at each stage were more than eight.

Published RNA sequencing (RNA-seq) results showed that Usp16 was the most abundantly expressed gene in human/mouse oocytes and early embryos among the genes encoding potential H2A DUBs (Figure 2A) (20,42). Quantitative RT-PCR (RT-qPCR) results showed that the expression level of murine *Usp16* was significantly higher in oocytes and early embryos than in other tissues, suggesting that maternal *Usp16* played an important role in MZT (Figure 2B). USP16 protein level remained stable during meiotic maturation (Figure 2C, E and F). Western blot and immunofluorescence results showed that the antibody was specific to endogenous USP16 (Supplementary Figure S4C and I) and USP16 was expressed in oocytes and continued to accumulate during early embryo development (Figure 2D-F). Some previous studies suggested that USP16 was cytoplasmic (43,44), while some claimed that USP16 was localized in the nucleus to perform the function of deubiquitination (27,37). Our observation was that USP16 mainly localized in the cytoplasm of oocytes and early embryos (Figure 2E and Supplementary Figure S4B-C). Only after GVBD, USP16 became distributed in the ooplasm evenly and had access to the chromatin (Figure 2E). In contrast, RNF2, the core component of PRC1, was primarily located in the nucleus at GV stage; it was distributed in the cytoplasm after nuclear envelop breakdown and gradually accumulated during oocyte maturation. After fertilization, RNF2 was further expressed and mainly localized to the nucleus (Figure 2C and Supplementary Figure S2). The expression pattern of RNF2 was consistent with the observation that H2AK119ub1 re-accumulated in zygotes and early embryos (Figure 1A, B).

### Changes in H2AK119ub1 status during oocyte-to-embryo transition

**Figure 3.**
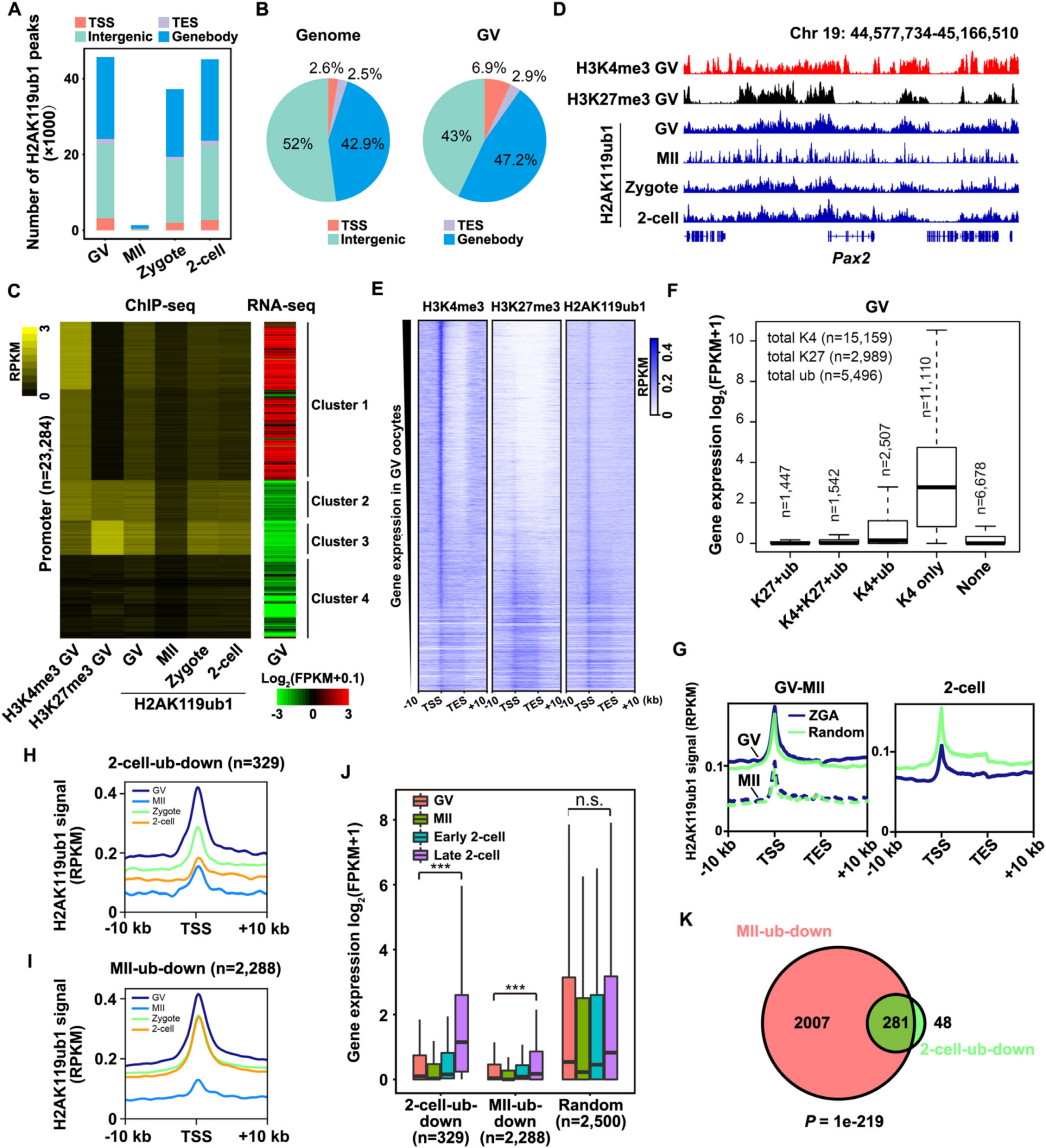
Changes in H2AK119ub1 status during oocyte-to-embryo transition. (**A**) Bar chart showing the number of H2AK119ub1 peaks located in TSS, TES, gene body and intergenic regions in oocytes and early embryos. (**B**) Genomic distribution of mouse genome (left) and H2AK119ub1 peaks in GV oocytes (right). (**C**) Heatmap showing histone modification enrichment at individual promoters in oocytes and early embryos, and gene expression levels in GV oocytes. Promoters were clustered into four groups using k-means clustering based on H3K4me (20)3 and H3K27me3 (62) signals in GV oocytes. (**D**) Genome browser view showing H3K4me3 and H3K27me3 enrichment in GV oocytes and H2AK119ub1 enrichment in oocytes and early embryos near *Pax2*. (**E**) Heatmap showing H3K4me3 (first column), H3K27me3 (second column) and H2AK119ub1 (third column) enrichment in individual genes in GV oocytes. Genes were sorted by expression level (FPKM). (**F**) Relationship between mRNA expression levels and enrichment of three histone modifications (H3K4me3, H3K27me3 and H2AK119ub1) on promoter regions in WT GV oocytes. Promoters were clustered into five groups using k-means clustering based on H3K4me3, H3K27me3 and H2AK119ub1 signals in GV oocytes. (**G**) Metaplot showing H2AK119ub1 enrichment of ZGA genes and random genes (identical numbers) in GV, MII oocytes and two-cell stage embryos. (**H**) Metaplot showing H2AK119ub1 enrichment of ‘two-cell-ub-down’ genes over promoter regions in oocytes and early embryos. (**I**). Metaplot showing H2AK119ub1 enrichment of ‘MII-ub-down’ genes over promoter regions in oocytes and early embryos. (**J**). Boxplot showing expression levels of ‘two-cell-ub-down’ and ‘MII-ub-down’ genes in oocytes and early embryos (20). The expression of genes randomly selected was as a control. The lower and upper hinges correspond to the first and third quartiles. Thick lines in boxes indicate the medians. The upper whisker extends from the hinge to the largest value no further than 1.5 × IQR from the hinge (where IQR is the inter-quartile range, or distance between the first and third quartiles). The lower whisker extends from the hinge to the smallest value at most 1.5 × IQR of the hinge. (**K**) Venn diagram showing overlap of ‘two-cell-ub-down’ and ‘MII-ub-down’ genes.

To study the role of H2AK119ub1 in early development in more detail, we determined the H2AK119ub1 enrichment profiles in oocytes and early embryos using ChIP-seq, and the replicates showed high correlations (Supplementary Figure S3A–C). We identified 45 698, 1354, 37 278 and 45 073 H2AK119ub1 peaks in GV oocytes, MII oocytes, zygotes and two-cell embryos, respectively (Figure 3A), consistent with our immunofluorescence results that H2AK119ub1 decreases during meiotic maturation and reaccumulates upon fertilization (Figure 1A, B). Compared to the genomic background, H2AK119ub1 is specifically enriched at the promoter regions (Figure 3B).

The H2AK119ub1 correlations for regions around TSSs were lower than that for regions around gene bodies among different stages, indicating that most dramatic changes occurred around the TSSs, where H2AK119ub1 levels were more enriched (Supplementary Figure S3B, C). Clustering all promoters according to their H3K4me3 (a hallmark for transcription initiation (45)) and H3K27me3 (which accumulated at a large cohort of silent genes(12)) enrichment in GV oocytes, and then plotting their H2AK119ub1 signals and corresponding gene expression levels(20) (Figure 3C), we found that promoter enrichment of H2AK119ub1 in GV oocytes largely resembled that of H3K27me3, which was more enriched in cluster 2–3 (silent genes) than cluster 1 (active genes) (Figure 3C, D). To our surprise, we also found the enrichment of H2AK119ub1 at the actively transcribed genes marked by H3K4me3 (20) (Figure 3C and E). So, we intended to further assess the relationship between co-occupancy of H2AK119ub1 with other two histone modifications and mRNA expression levels (20) of GV oocytes (Figure 3F and Supplementary Figure S3D). Clustering results revealed 5496 genes with H2AK119ub1-enriched promoters in the GV oocytes, which were more abundant than the genes with H3K27me3-enriched promoters (*n* = 2989), but less abundant than H3K4me3-enriched promoters (*n* = 15 159) (Figure 3F). Genes with only H3K4me3 at promoters (*n* = 11 110) were the most active in GV oocytes (Figure 3F, lane 4). Among these H3K4me3-modified genes, those also modified by H2AK119ub1 appeared to have relatively lower expression levels (20) (Figure 3F, lane 3), indicating that H2AK119ub1 potentially played an inhibitory role in transcription regulation. H2AK119ub1 usually coexisted with other modifications and often worked along with H3K27me3 to inhibit transcription, which had the strongest inhibiting ability (Figure 3F, lanes 1–2).

ZGA at the two-cell stage was vital for embryogenesis and the generated RNAs at that corresponding stage were remarkably different from those generated in oocytes (46–49). We have examined the H2AK119ub1 signals at ZGA genes (i.e. genes with FC [two-cell/zygote] > 3) and random genes (a certain number of randomly selected genes) from GV to two-cell stage with our and the public ChIP-seq data. H2AK119ub1 signals at ZGA and random genes were both decreased at MII stage. Until two-cell stage, H2AK119ub1 signals at random genes were reconstructed and much higher than that at ZGA genes (Figure 3G and Supplementary Figure S3E), indicating H2AK119ub1 removal during oocyte maturation might be vital for proper zygotic H2AK119ub1 reconstruction and ZGA in early embryos. We next identified 329 genes with significant decrease of promoter H2AK119ub1 between GV oocytes and two-cell embryos as ‘two-cell-ub-down’ genes, and similarly identified 2,288 genes with significant decrease of promoter H2AK119ub1 between GV and MII oocytes as ‘MII-ub-down’ genes (Figure 3H-I). Compared with random genes, we found that both ‘two-cell-ub-down’ and ‘MII-ub-down’ genes were relatively more active in two-cell embryos (20) (Figure 3J). Furthermore, H2AK119ub1 levels of most (281/329) ‘two-cell-ub-down’ genes already decreased at the MII stage (Figure 3K). Collectively, these results indicated that maternal H2AK119ub1 removal after meiosis resumption might be required to maintain low H2AK119ub1 levels at the promoters of early zygotic genes, and a prerequisite for their activation in two-cell embryos.

### Meiosis-coupled histone H2AK119 deubiquitination does not occur in Usp16-null oocytes

To investigate the potential role of USP16 in regulating H2AK119ub1, as well as the physiological significance of this regulation in MZT, we performed conditional knockout (cKO) of USP16 in mouse oocytes at as early as the primordial follicle stage using *Gdf9-Cre* (Supplementary Figure S4A). Efficient USP16 protein depletion in oocytes was confirmed using immunohistochemistry, immunofluorescence and western blot (Supplementary Figure S4B, C and I). *Usp16^fl/fl^;Gdf9-Cre* adult females displayed normal ovarian histology (Supplementary Figure S4D). Ovaries of pubertal *Usp16^fl/fl^;Gdf9-Cre* mice (3-week-old) contained multiple growing antral follicles 44 h after pregnant mare serum gonadotropin (PMSG) injection (Supplementary Figure S4E, upper panels). After human chorionic gonadotropin (hCG) injection, *Usp16^fl/fl^;Gdf9-Cre* females ovulated normally (Supplementary Figure S4E, lower panels). Similar numbers of ovulated oocytes were observed in the oviducts of WT and *Usp16^fl/fl^;Gdf9-Cre* females, whether pubertal females or adult females (Supplementary Figure S4F). The ovulated *Usp16*-null oocytes could develop to the MII stage; each contained a normal-shaped spindle and was attached to an emitted polar body-1 (PB1, Supplementary Figure S4G-H). Maternal proteins important for female fertility, including RNF2, ZAR1 and BTG4, had normal expression patterns, as seen in WT oocytes (Supplementary Figure S4I). These results indicated that USP16 is dispensable for meiotic cell cycle progression in oocytes.

**Figure 4.**
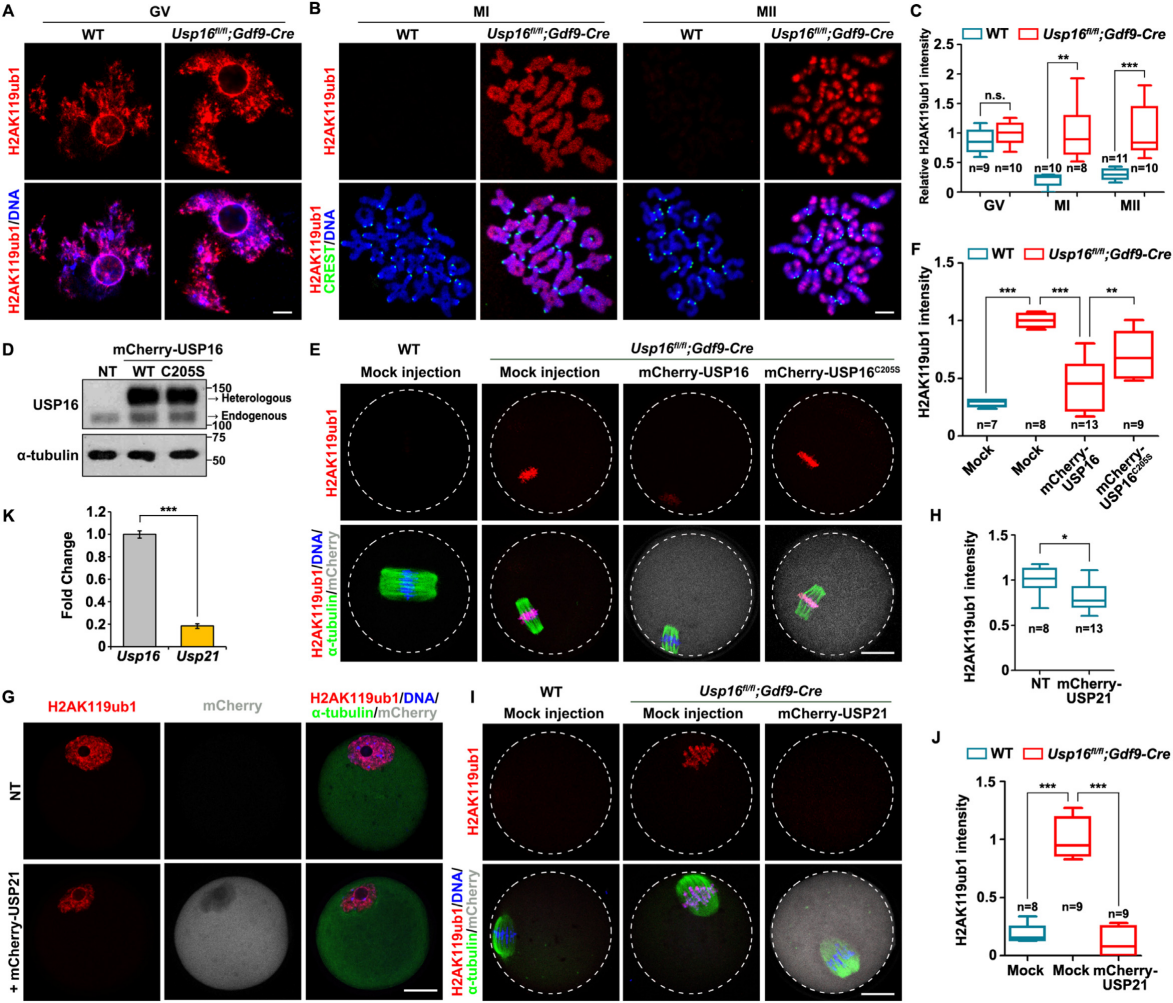
Maternal USP16 deletion impaired H2AK119ub1 decline after meiosis resumption. (A, B) Levels of H2AK119ub1 in GV (**A**), MI and MII (**B**) oocytes of WT and *Usp16^fl/fl^;Gdf9-Cre* females. Centromeres and DNA were labeled using CREST (green) and DAPI (blue), respectively. Scale bar, 5 μm. (**C**) Quantification of H2AK119ub1 signal intensity of (A) and (B). The numbers of analyzed oocytes at each stage were indicated. Error bars, SEM. **P< 0.01, ***P< 0.001 by two-tailed Student's *t*-test. n.s.: non-significant. (**D**) Western blot results showing the overexpression of active/inactive USP16 in oocytes after mRNA microinjection. Total proteins from 100 oocytes were loaded in each lane. α-tubulin was blotted as a loading control. (**E**) Immunofluorescence showing levels of H2AK119ub1 of WT and *Usp16^fl/fl^;Gdf9-Cre* oocytes, as well as *Usp16^fl/fl^;Gdf9-Cre* oocytes overexpressing USP16 and USP16^C205S^. All the oocytes were analyzed at the MI stage. Scale bar, 20 μm. (**F**) Quantification of H2AK119ub1 signal intensity of (E). Error bars, SEM. **P< 0.01, ***P< 0.001 by two-tailed Student's *t*-test. (**G**) Immunofluorescence of H2AK119ub1 in GV oocytes with/without exogenous USP21. Scale bar, 20 μm. (**H**) Quantification of H2AK119ub1 signal intensity of **(G**). Error bars, SEM. *P< 0.05 by two-tailed Student's *t*-test. (**I**). Immunofluorescence showing levels of H2AK119ub1 in WT and *Usp16^fl/fl^;Gdf9-Cre* oocytes, as well as *Usp16^fl/fl^;Gdf9-Cre* oocytes overexpressing USP21. Scale bar, 20 μm. (**J**) Quantification of H2AK119ub1 signal intensity of (**I**). Error bars, SEM. ***P< 0.001 by two-tailed Student's *t*-test. (**K**) RT-qPCR results showing the relative expression levels of *Usp16* and *Usp21* in oocytes. *n* = 3 biological replicates. Error bars, SEM. ***P< 0.001 by two-tailed Student's *t*-test.

Immunofluorescence analysis indicated that the H2AK119ub1 level was not altered in the fully grown oocytes isolated from antral follicles of *Usp16^fl/fl^;Gdf9-Cre* females (Figure 4A and C). However, H2AK119ub1 failed to be removed from the condensed chromosomes in *Usp16*-null oocytes at the MI and MII stages (Figure 4B and C). In a rescue experiment, ectopic expression of mCherry-USP16 in *Usp16*-null oocytes by mRNA microinjection could not remove H2AK119ub1 from the chromosomes at GV stage, as we speculated (Supplementary Figure S5A-B), but could at MI stage (Figure 4D–F). Cysteine-205 of USP16 was vital for its deubiquitinase activity; as a negative control, when mCherry-USP16^C205S^ was expressed in *Usp16*-null oocytes, it failed to remove H2AK119ub1 from the chromosomes (Figure 4D–F), indicating that the deubiquitinase activity of USP16 is essential for H2AK119ub1 removal in maturing oocytes.

We then asked whether the H2AK119 deubiquitination activity of USP16 is unique or it can be substituted by other H2AK119ub1-targeted DUBs. We compared the expression levels of *Usp16* and *Usp21* in GV oocytes by absolute RT-qPCR and found *Usp16* was more than 5-fold higher than that of *Usp21* (Figure 4K). And deletion of *Usp16* did not substantially change the expression levels of mRNAs encoding other DUBs (Supplementary Figure S5C). We introduced ectopic expression of mCherry-USP21 in GV oocytes by microinjection and observed that USP21 also localized in the ooplasm and decreased the H2AK119ub1 level slightly (Figure 4G-H). Similar to USP16, however, overexpression of USP21 in *Usp16*-null oocytes was able to decline the H2AK119ub1 signals on condensed chromosomes after meiotic resumption (Figure 4I, J). These results indicate that besides USP16, there were other DUBs that possessed H2AK119 deubiquitination activity in oocytes, but their protein levels were normally too low to support their function.

It has been reported in cultured somatic cell lines that USP16-dependent H2AK119 deubiquitination is a prerequisite for histone H3 serine-10 phosphorylation (H3S10ph) during mitosis entry(27). However, in *Usp16*-null MI oocytes, H3S10ph levels were comparable to that in WT oocytes at the same stage (Supplementary Figure S5D, E).

### *Usp16* knockout affects H2AK119ub1 distribution after meiosis resumption

**Figure 5.**
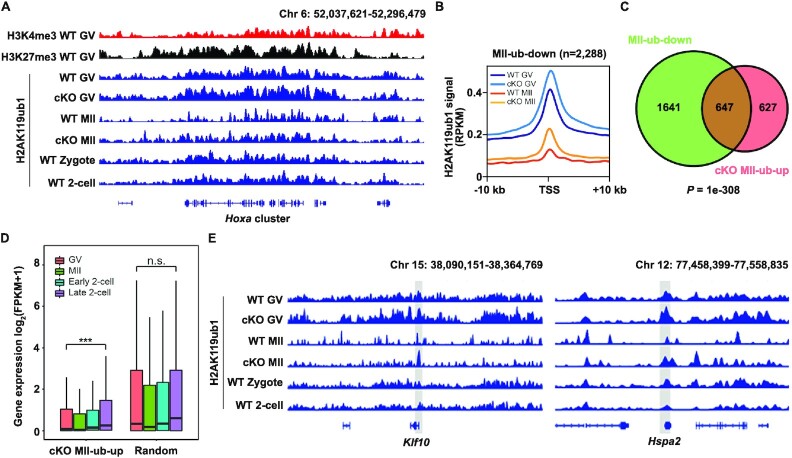
Impact of maternal *Usp16* knockout on genomic H2AK119ub1 distribution. (**A**) The genome browser view showing H3K4me3, H3K27me3 and H2AK119ub1 near *Hoxa* cluster at indicated developmental stages. (**B**) Metaplot showing H2AK119ub1 enrichment of ‘MII-ub-down’ genes over promoter regions in WT and *Usp16^fl/fl^;Gdf9-Cre* oocytes. (**C**) Venn diagram showing overlapping of ‘MII-ub-down’ and ‘cKO MII-ub-up’ genes. (**D**) Boxplot showing expression levels of ‘cKO MII-up’ genes in oocytes and early embryos (20). (**E**) The genome browser view showing H2AK119ub1 enrichment near *Klf10* and *Hspa2* at indicated developmental stages.

To determine the genome-wide H2AK119ub1 changes after maternal *Usp16* knockout, we performed H2AK119ub1 ChIP-seq with GV and MII oocytes of WT and *Usp16^fl/fl^;Gdf9-Cre* mice. Consistent with the immunofluorescence results, *Usp16* knockout led to high level of H2AK119ub1 at MII stage (Figure 5A). And for the previously described ‘MII-ub-down’ genes, H2AK119ub1 was indeed retained around their TSS in *Usp16*^−^*^/^*^−^ MII oocytes (Figure 5B and E). We identified 1274 genes with promoter H2AK119ub1 levels up-regulated in *Usp16*^−^*^/^*^−^ MII oocytes as ‘cKO MII-ub-up’ genes, 51% of which belonged to ‘MII-ub-down’ genes (Figure 5C). ‘CKO MII-ub-up’ genes were generally activated during normal ZGA (Figure 5D). We also found that maternal USP16 deletion disrupted the deubiquitination of *Klf10* and *Hspa2*, two ZGA genes, at their promoter regions, which might lead to the failure of ZGA (Figure 5E). In sum, these results further suggested that maternal H2AK119ub1 removal by USP16 during oocyte maturation might be vital for proper zygotic H2AK119ub1 reconstruction and ZGA in early embryos. This hypothesis would be further examined in the following experiments.

### Maternal *Usp16*-deleted two-cell embryos display ZGA defects

Though H2AK119ub1 level was high in *Usp16*-null MII oocytes, it seemed comparable in WT and maternal *Uspl6* knockout (cKO) zygotes (Supplementary Figure S6A-B). It may well be that the enrichments of H2AK119ub1 at individual gene were different and the difference could not be observed simply by immunofluorescence. To investigate the direct or indirect effects of maternal *Usp16* deletion on ZGA, we subjected samples of zygotes and two-cell stage embryos of both genotypes to RNA-seq analyses, and all replicates showed high correlations (Supplementary Figure S6C).

**Figure 6.**
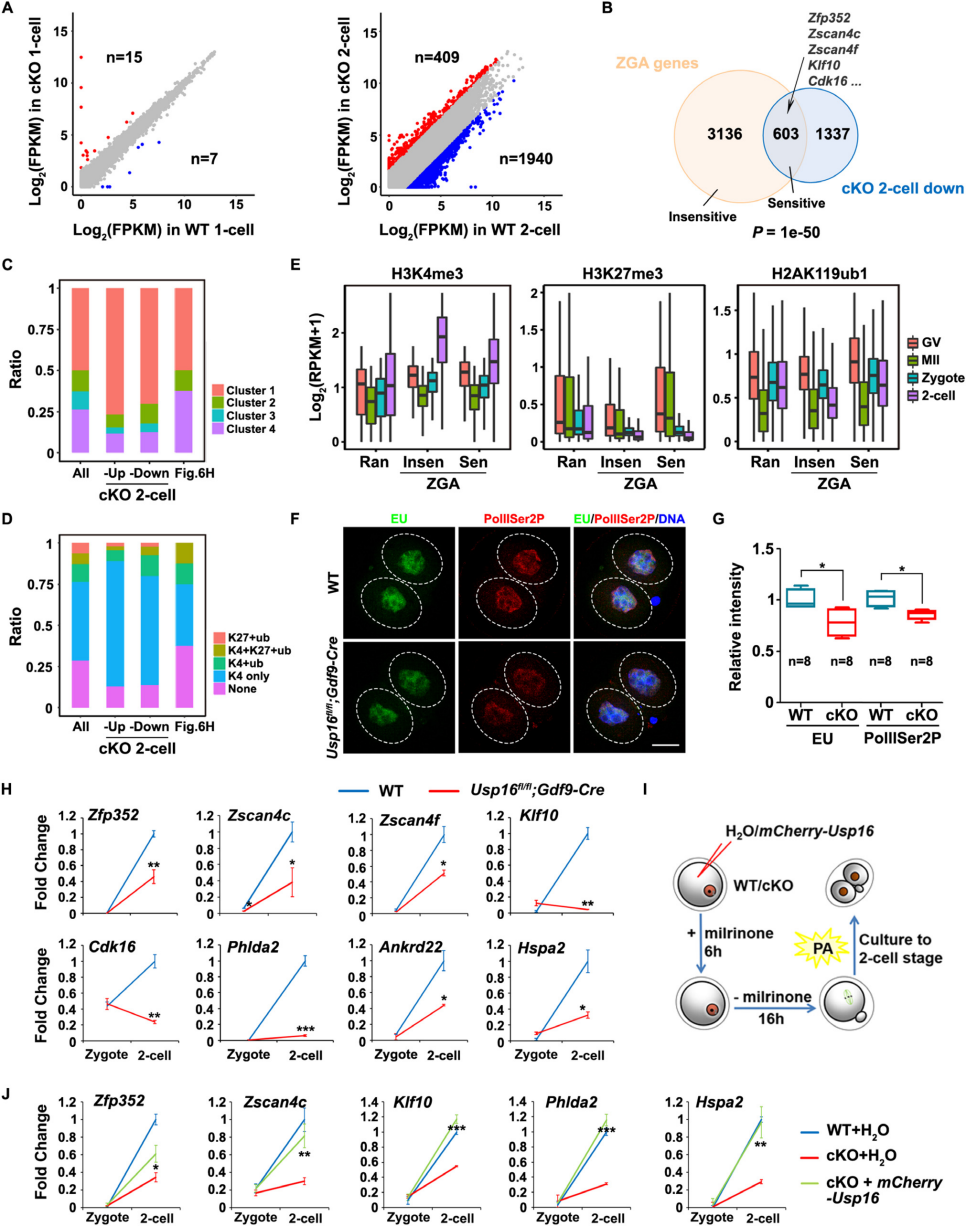
USP16 deletion in oocytes caused ZGA failure in the two-cell embryos. (**A**) Scatter plot comparing the transcripts of WT and maternal *Usp16* knockout embryos (zygotes and two-cell stage embryos). Transcripts that increased or decreased by more than 3-fold in maternal *Usp16* knockout embryos are highlighted in red or blue, respectively. (**B**) Venn diagrams showing the overlap of transcripts that were significantly degraded in maternal *Usp16* knockout two-cell stage embryos and ZGA genes. (**C**, **D**). Bar plot showing enrichment of all, ‘cKO two-cell up’ and ‘cKO two-cell down’ genes from RNA-seq data and the genes from (H) in clusters of Figure 3C (C) and F (D). (**E**) Boxplot showing H3K4me3/H3K27me3/H2AK119ub1 enrichment at USP16-insensitive and -sensitive ZGA genes at indicated stages. ‘Ran’, ‘Insen’ and ‘Sen’ represented random genes, USP16-insensitive ZGA genes and USP16-sensitive ZGA genes, respectively. (**F**) Levels of EU and PolIISer2P in two-cell embryos of WT and *Usp16^fl/fl^;Gdf9-Cre* females. Scale bar, 20 μm. (**G**) Quantification of EU and PolIISer2P signal intensity in two-cell stage embryos of WT and *Usp16^fl/fl^;Gdf9-Cre* females. The numbers of analyzed embryos were indicated (n). Error bars, SEM. *P< 0.05 using two-tailed Student's *t*-test. (**H**) RT-qPCR results showing the relative levels of indicated transcripts in zygotes and two-cell embryos of WT and *Usp16^fl/fl^;Gdf9-Cre* females. n = 3 biological replicates. Error bars, SEM. *P< 0.05, **P< 0.01, ***P< 0.001 using two-tailed Student's *t*-tests. (**I**) Schematic diagram showing the generation of parthenogenetic embryos with different genotypes as well as USP16 over-expression. (**J**). RT-qPCR results showing the relative levels of indicated transcripts in zygotes and two-cell embryos of WT and *Usp16^fl/fl^;Gdf9-Cre* females, as well as *Usp16^fl/fl^;Gdf9-Cre* oocytes overexpressing USP16 *in vitro*. n = 3 biological replicates. Error bars, SEM. *P< 0.05, **P< 0.01, ***P< 0.001 using two-tailed Student's *t*-test.

Only 15 and 7 transcripts were up-regulated [fold change (cKO/WT) > 3] and down-regulated [fold change (WT/cKO) > 3] in the maternal *Uspl6* knockout zygotes (Figure 6A, left panel). At the two-cell stage, 1940 transcripts were down-regulated (Figure 6A, right panel), 31% of which were ZGA genes (Figure 6B), indicating the failure of ZGA. Both ‘cKO two-cell down/up’ genes were similarly enriched in the clusters with high levels of H3K4me3, relatively low levels of H2AK119ub1 and H3K27me3 (Figure 6C, D). Compared to up-regulated genes, ‘cKO two-cell down’ genes were more enriched in H2AK119ub1 demarcated clusters (K4 + K27 + ub and K4 + ub), indicating the genes with more H2AK119ub1 enrichment at GV stage may be more sensitive to USP16 and down-regulated when ZGA happened without H2A deubiquitination (Figure 6C, D). We divided ZGA genes into USP16-insensitive and -sensitive ZGA genes (Figure 6B) and found H2AK119ub1 signals at USP16-sensitive ZGA genes were higher than those at USP16-insensitive ZGA genes at the GV stage in both our and the public ChIP-seq data (Figure 6E, right panel and Supplementary Figure S6D). USP16-sensitive ZGA genes also showed lower H3K4me3 enrichment at the two-cell stage (Figure 6E, left panel) and higher H3K27me3 enrichment at the GV and MII stage (Figure 6E, middle panel) than USP16-insensitive ZGA genes. Taken together, USP16-sensitive ZGA genes may have higher repressive histone modifications in oocytes. Besides, the down-regulated ZGA genes had higher fold change of H2AK119ub1 levels than up-regulated ZGA genes at the MII stage after *Usp16* deletion (Supplementary Figure S6E). H2AK119ub1 level of non-differentially expressed ZGA genes also increased as down-regulated ZGA genes in *Usp16*^−^*^/^*^−^ MII oocytes, implying the transcriptional inhibition role of H2AK119ub1 might not be very strict, or these genes might be simultaneously regulated by H2AK119ub1 and other modifications (Supplementary Figure S6E). Conversely, ZGA genes that retained higher H2AK119ub1 levels at the MII stage after *Usp16* deletion had more severe defects in gene expression at the two-cell stage (Supplementary Figure S6F). These results further confirmed that the retention of H2AK119ub1 after meiosis resumption was associated with impaired ZGA. H2AK119ub1 removal during meiosis controlled the transcriptional responses at two-cell stage both directly or indirectly.

We further analyzed the transcriptional activity in two-cell embryos generated after maternal *Usp16*-deletion. To label the newly synthesized RNAs, EU was added to the medium 1 h before fixation of control and cKO embryos. Weaker EU signals were detected in cKO embryos (Figure 6F, G). This observation was consistent with the immunofluorescence staining result of phosphorylated RNA polymerase II at serine-2 (PolIISer2P, a marker of RNA polymerase II activation) (Figure 6F-G). The impaired ZGA in cKO embryos was validated by the RT-qPCR results, which demonstrated that known early zygotic genes were expressed at compromised levels at the two-cell stage (Figure 6H). These genes included *Zscan4* family members, which maintain totipotency in two-cell embryos and two-cell-like stem cells(50,51); *Klf10*, which was transcription related (52); as well as other early zygotic genes (*Zfp352*, *Cdk16*, *Phlda2*, *Ankrd22* and *Hspa2*) with undetermined functions in maternal zygotic transition. Among these, *Klf10* and *Hspa2* belonged to the group of ‘MII-ub-down’ genes and ‘cKO MII-ub-up’ genes and *Hspa2* also belonged to ‘two-cell-ub-down’ genes (Figure 5E). These results indicated that ZGA was impaired by maternal *Usp16* deletion directly or indirectly.

To determine whether the impaired ZGA was directly caused by the absence of H2AK119ub1 deubiquitination after *Usp16* deletion, we overexpressed mCherry-USP16 in fully grown *Usp16*-null oocytes by mRNA microinjection. Sixteen hours after the oocytes were released from milrinone, mature MII oocytes were then subjected to parthenogenetic activation (PA) treatment. WT and *Usp16*-null oocytes injected with H_2_O were used as controls. In this experiment, PA, instead of *in vitro* fertilization (IVF), was used to induce ZGA because the *in vitro* cultured and microinjected oocytes usually had low fertilization rates via IVF. After another 30 h culture *in vitro*, parthenogenetic two-cell embryos were collected for detecting the expression of early zygotic genes using RT-qPCR, as illustrated in Figure 6I. The exogenous expression of catalytically active/inactive USP16 in WT oocytes did not affect the timing of embryo development (Supplementary Figure S6G, H), so we could conduct this experiment. Similar to the results in *in vivo* fertilized two-cell embryos, these genes were transcriptionally activated in parthenogenetic *Usp16^♀^^+^* embryos but were at lower levels in *Usp16^♀−^* embryos. Ectopic USP16 expression partially restored the expression of these early zygotic genes (Figure 6J), suggesting that USP16-mediated H2AK119ub1 deubiquitination are important for ZGA.

### Maternal USP16 deletion in mice causes female subfertility

**Figure 7.**
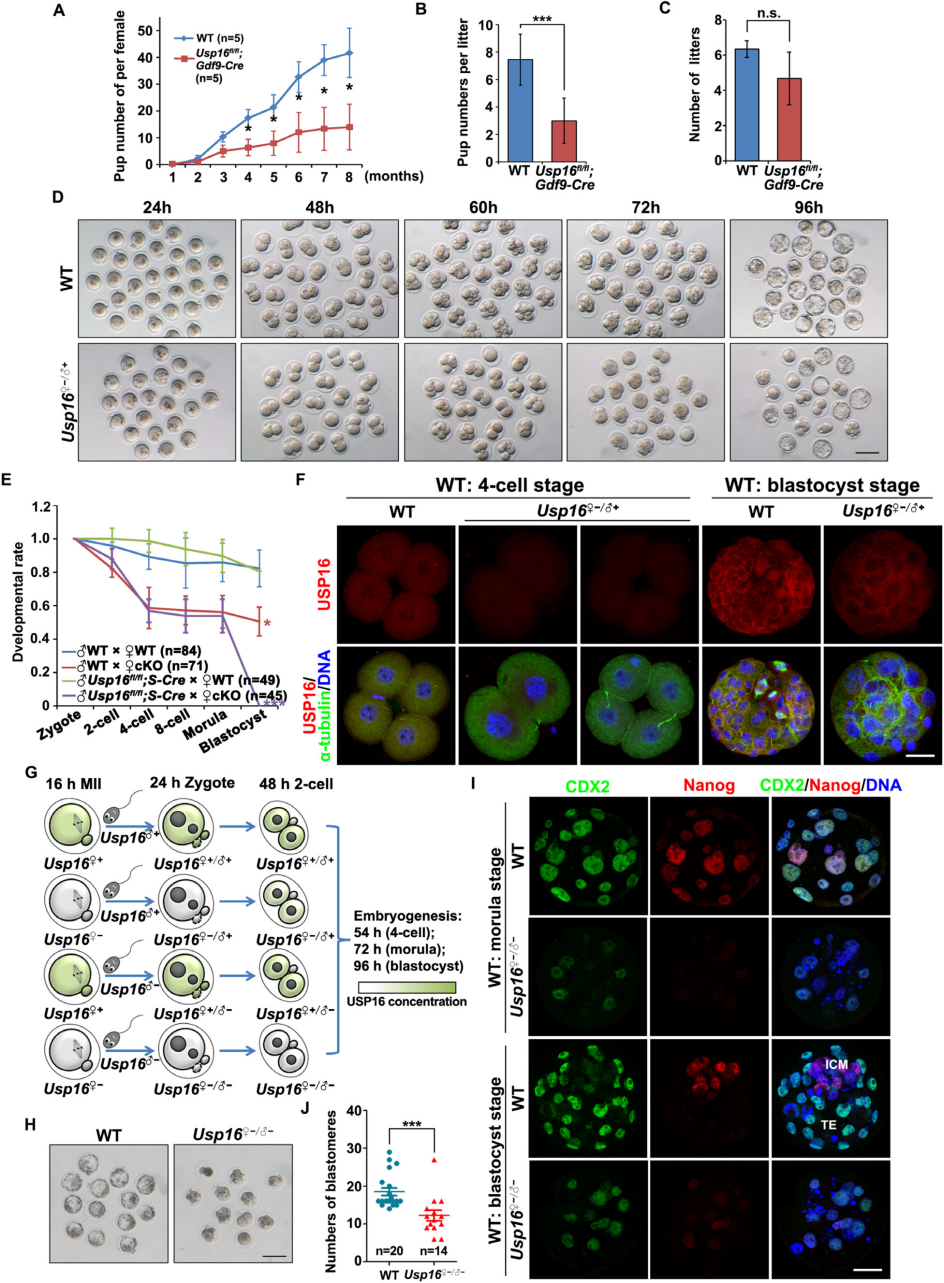
Maternal deletion of USP16 caused female subfertility owing to impaired early embryogenesis. (**A**) Cumulative pup numbers per female WT and *Usp16^fl/fl^;Gdf9-Cre* mice. The numbers of analyzed mice have been indicated (n). Error bars, S.E.M. *P< 0.05 using two-tailed Student's *t*-test. (**B**) Number of pups per litter. Error bars, S.E.M. ***P< 0.001 using two-tailed Student's *t*-test. (**C**) Number of litters per female mouse during eight months for fertility tests. Error bars, S.E.M. n.s.: non-significant. (**D**) Representative images of preimplantation embryos derived from WT and *Usp16^fl/fl^;Gdf9-Cre* females at the time when WT embryos reached the indicated stages. ‘♂’ and ‘♀’ were used to refer to paternal and maternal alleles, respectively. Scale bar, 100 μm. (**E**) Quantification of preimplantation embryos of different genotypes at the time when WT embryos reached the indicated stages. The numbers of analyzed embryos have been indicated (n). Error bars, S.E.M. *P< 0.05, ***P< 0.001 using two-tailed Student's *t*-test. (**F**) Immunofluorescent staining showing the expression of USP16 in WT and *Usp16^♀−^^/^^♂^^+^* embryos. Scale bar, 20 μm. (**G**) Schematic diagram showing the generation of embryos by parents of different genotypes. (**H**). Representative images of WT and *Usp16^♀−^^/^^♂−^* embryos at 104 h after hCG injection. (**I**) Immunofluorescence of CDX2 and Nanog in embryos at the time when WT embryos developed to the morula and blastocyst stages. Scale bar, 20 μm. (**J**) The number of blastomeres per WT or *Usp16^♀−^^/^^♂−^* embryo at 80 h after hCG injection, when the WT embryos developed to the morula stage. The numbers of analyzed embryos have been indicated (n). Error bars, S.E.M. ***P< 0.001 using two-tailed Student's *t*-test.

To determine the *in vivo* significance of USP16-mediated H2AK119 deubiquitination during MZT, we analyzed the fertility of *Usp16^fl/fl^;Gdf9-Cre* females by crossing them with adult WT males. In the 8-month fertility test, *Usp16^fl/fl^;Gdf9-Cre* females showed reduced fertility (Figure 7A); the litter size was significantly smaller than that of WT females (Figure 7B), but the average number of litters per female was close to normal levels (Figure 7C). We then studied the development of zygotes derived from *Usp16^fl/fl^;Gdf9-Cre* females. Superovulated *Usp16^fl/fl^;Gdf9-Cre* females were crossed with fertile WT males, and successful coitus was confirmed by the presence of vaginal plugs. The resulting maternal *Usp16*-deleted embryos (*Usp16^♀−^^/^^♂^^+^*) had a prolonged two-cell stage compared to that of WT controls (*Usp16^♀^^+/^^♂^^+^*) and only 50% of them could develop to the four-cell stage. However, most *Usp16^♀−^^/^^♂^^+^* embryos that had escaped from the two-cell arrest could successfully develop to the blastocyst stage (Figure 7D-E). Because the paternal *Usp16* allele was intact in *Usp16^♀−^^/^^♂^^+^* embryo and re-express at two-cell stage (Supplementary Figure S6I), we detected the expression of USP16 in these embryos by immunofluorescence. USP16 signal was undetectable in *Usp16^♀−^^/^^♂^^+^* embryos at the four-cell stage but was present in those that developed into blastocysts (Figure 7F). These results suggested that the expression of paternal USP16 after the two-cell stage partially rescued the developmental defects in *Usp16^♀−^^/^^♂^^+^* embryos.

To test this hypothesis, we developed *Usp16^fl/^^−^;Stra8-Cre* male mice, with *Usp16* knockout in the male germline (Figure 7G). When *Usp16^fl/fl^;Gdf9-Cre* females were mated with these males, the resultant *Usp16^♀−^^/^^♂−^* embryos exhibited severe developmental defects because of ablated USP16 expression (Figure 7E and G). Similar to the *Usp16^♀−^^/^^♂^^+^* embryos, 50% *Usp16^♀−^^/^^♂−^* embryos were blocked at the two-cell stage. However, the embryos that escaped from the two-cell arrest were blocked at the morula stage (Figure 7E and H). Only a few blastomeres of the *Usp16^♀−^^/^^♂−^* embryos expressed caudal type homeobox 2 (CDX2, a functional marker for trophectoderm (TE)) and Nanog (a functional marker for inner cell mass (ICM)), indicating that the blastomeres in these developmentally arrested embryos failed to acquire a TE or an ICM cell fate (Figure 7I). Besides, many blastomeres in *Usp16^♀−^^/^^♂−^* embryos had fragmented nuclei (Figure 7I), suggesting potential roles of USP16 in genome stability maintenance during embryogenesis. Though the developmentally arrested *Usp16^♀−^^/^^♂−^* embryos appeared compacted (Figure 7H), the number of blastomeres of these embryos was fewer than that of WT morula (Figure 7J). These results indicated that: (i) maternal USP16 deletion caused partial early developmental arrest after fertilization; and (ii) zygotically expressed USP16 quickly substituted for maternal USP16 in supporting embryo development beyond the morula stage.

## DISCUSSION

Studies in model systems including *Drosophila*, zebrafish, *Xenopus* and mouse have indicated that H2AK119ub1 is a fundamental epigenetic marker that controls many crucial cellular processes in development and homeostasis maintenance (53,54). Mammalian oocytes and embryos also undergo drastic epigenetic changes during development (2,5). Like other histone modifications, H2AK119ub1 is a reversible mark. Its levels are determined by a balance between PRC1-mediated H2AK119 ubiquitination and H2AK119 DUB-mediated deubiquitination (55). However, neither the significance of H2AK119ub1 during the MZT process, nor the potential H2AK119 DUB has been verified in mammalian oocytes or early embryos.

**Figure 8.**
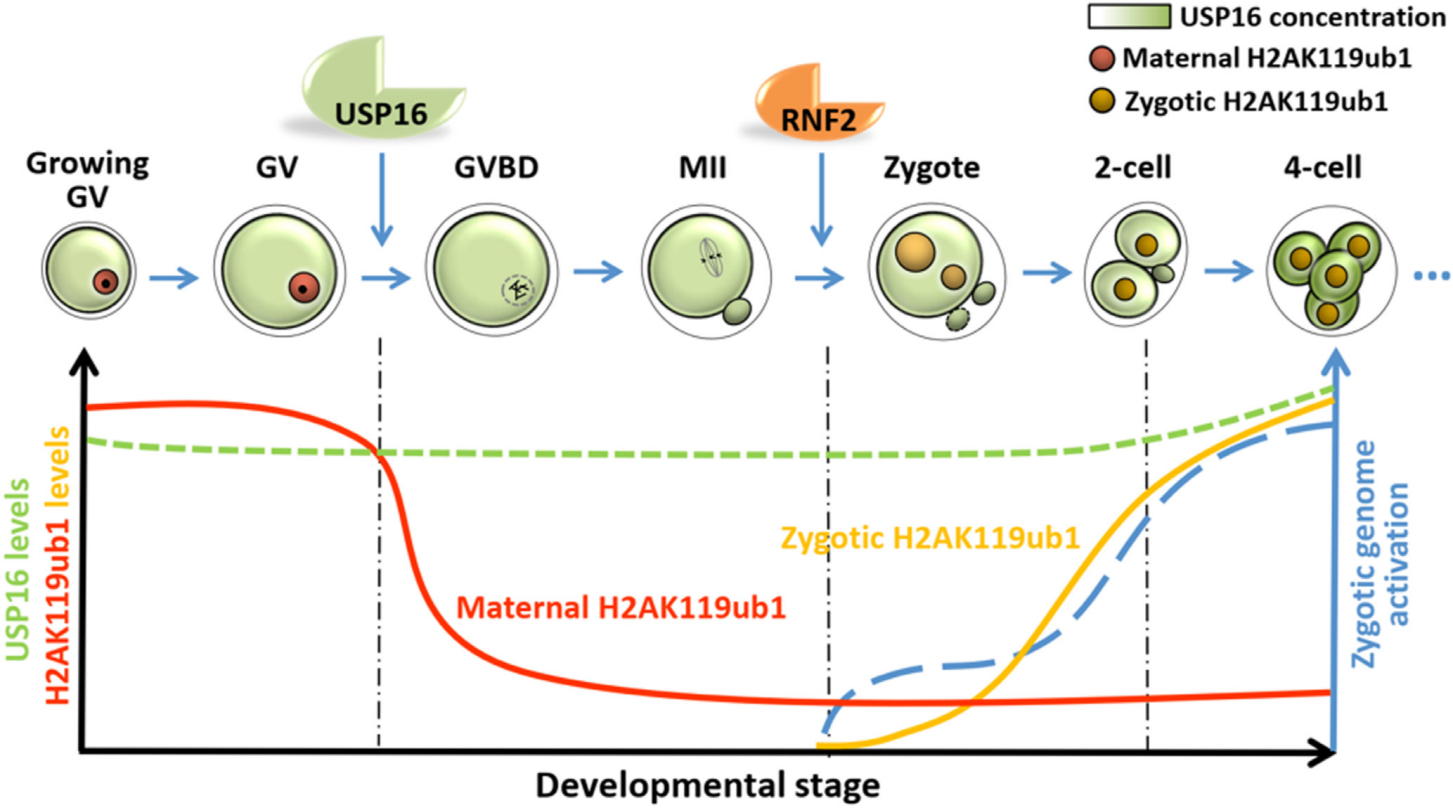
A diagram showing the significance of H2AK119ub1 removal by USP16 during MZT. During follicle growth, H2AK119ub1 was maintained at a high level by PRC1 in the oocytes. A H2AK119 deubiquitinase USP16 was expressed in the ooplasm but did not have access to the chromatin. At meiosis resumption, maternal H2AK119ub1 was deubiquitinated by USP16 soon after nuclear membrane breakdown to prepare for epigenetic reprogramming. After fertilization, maternal RNF2 protein stored in the ooplasm returned to nucleus to mediate reconstruction of zygotic H2AK119ub1. The deletion of maternal *Usp16* had little effect on oocyte growth and meiosis, but caused maternal H2AK119ub1 not to be removed during meiosis and to be abnormal at individual ZGA genes in subsequent embryos. USP16 ensured zygotic genome reprogramming and embryonic development in order.

Previously, maternal H2AK119ub1 was found to be maintained at a high level by PRC1 in GV oocytes and undetectable in MII oocytes (16,17). Then H2AK119ub1 continued to be present in the pronuclei of zygotes and nuclei of preimplantation embryos (18). Our H2AK119ub1 immunofluorescence results are consistent with these observations. However, we further detected the prompt decrease in H2AK119ub1 in the time frame following meiotic resumption, indicating that the removal of H2AK119ub1 could be a novel epigenetic marker of meiotic resumption. The present study revealed that the key DUB in oocytes was USP16, which was localized in the ooplasm before meiotic resumption. Upon nuclear envelope breakdown, USP16 acquired access to chromatin to remove H2AK119ub1. At the same time, components of the nuclear factor PRC1 were released into the cytoplasm and could not catalyze ubiquitylation of histone H2A possibly because the chromatin was condensed at this state. After fertilization, the chromatin became decondensed and the PRC1 components reloaded on the chromatin of both the pronuclei to generate embryonic H2AK119ub1, which was maintained at a higher level during subsequent embryo development (Figure 8).

During oogenesis, PRC1 repressed aberrant transcription to specify maternal factors in the cytoplasm and on chromosomes to contribute to the embryo developmental potential (17). RNF2 and its paralog RING1, the two core PRC1 components, are the main enzymes responsible for H2AK119ub1 in oocytes (17). Maternal deficiency for *Rnf2* and *Ring1* resulted in undetectable H2AK119ub1, massive transcriptional misregulation in oocytes, impaired ZGA, and embryo development blocking at the two-cell stage that followed (17). Nevertheless, it is not clear whether PRC1 works that way by blocking the elongation of RNA polymerase through H2AK119ub1 or not, because non-enzymatic PRC1 could also repress transcription by compacting chromatin directly, blocking remodeling and inhibiting transcription initiation (56). In a recent study, Jullien *et al.* used the nuclear transfer strategy to identify genes in MEF nuclei whose epigenetic configuration directly resisted transcriptional reprogramming (10). Overexpression of the chromatin modifier USP21, another H2AK119ub1 DUB, in advance could reduce the H2AK119ub1 level, alleviate transcriptional reprogramming resistance, and promote embryo development after nuclear transplantation (10), suggesting that the destabilization of innate epigenetic repression on genes critical for safeguarding the identity of a cell was indispensable to the reestablishment of gene expression patterns. Identifying definitive evidence that proves the function of H2AK119ub1 in transcriptional repression *in vivo* has remained a challenge in oocytes and early embryos. In our study, we first determined the significance of H2AK119ub1 and its reduction in ZGA *in vivo*. Defective H2AK119ub1 deubiquitination after GVBD by maternal *Usp16* knockout did not affect meiosis, but impaired ZGA and subsequent early embryonic development, due to the failure of zygotic genome reprogramming. Overall, the proper establishment and timely removal of H2AK119ub1 in oocytes were both vital for early embryo development.

Further insight into the role of H2AK119ub1 came from the findings of ChIP-seq studies. H2AK119ub1 specifically deposited at gene promoters in oocytes and embryos, implying its role in transcriptional regulation. By comparing the ChIP-seq results with the RNA-seq results at the GV stage, it was possible to gain insight into the negative correlation between H2AK119ub1 levels and gene expression. Although H2AK119ub1 existed in the entire genome, it preferred to accumulate at the genes with low expression levels.

It has been reported that multiple epigenetic marks localize on distinct genomic regions and regulate transcription at a single-gene level (57,58). Therefore, we checked the correlation of H2AK19ub1 with other histone modifications in oocytes (Figure 3C–F). H2AK119ub1 tended to be deposited at both H3K27me3-enriched domains and H3K4me3-enriched domains, indicating that H2AK119ub1 indeed preferred to function together with other histone modifications. The genes enriched by H3K4me3 usually had high expression level and the genes enriched by H3K27me3 usually had low expression level, consistent with their transcriptional regulation activity. The genes enriched by H2AK119ub1 and H3K4me3 had lower expression levels than those enriched only by H3K4me3, indicating that H2AK119ub1 potentially played an inhibitory role in transcription regulation. Moreover, the expression levels of the genes enriched by H2AK119ub1 and H3K27me3, as well as the genes enriched by H2AK119ub1, H3K4me3 and H3K27me3 were much lower. In conclusion, a variety of histone modifications cooperated to regulate gene transcription in mouse oocytes.

By analyzing RNA-seq result, we found 409 and1940 transcripts were up- and down-regulated respectively in cKO two-cell stage embryos. Though all the changed genes preferred to belong to the clusters with high levels of H3K4me3, relatively low levels of H2AK119ub1 and none H3K27me3, there were more H2AK119ub1 enriched clusters (K4 + K27 + ub and K4 + ub) in down-regulated genes (Figure 6C-D), indicating the genes with more H2AK119ub1 at GV stage were more sensitive and down regulated when ZGA happened without deubiquitination during meiosis. H2AK119ub1 removal during meiosis controlled the transcriptional responses at two-cell stage in a direct or indirect manner. As for genes in Figure 6H, the number was too little to perform such enrichment analysis or to get meaningful results (Figure 6C, D). Besides, only 9.4% of 329 ‘two-cell-ub-down’ genes and 7.6% of 2288 ‘MII-ub-down’ genes were down-regulated (data not shown) in maternal *Usp16*-deleted two-cell embryos, suggesting that more ZGA genes were regulated by USP16 indirectly. However, almost all these down-regulated ‘two-cell-ub-down’ genes (90.0%) were included in these down-regulated ‘MII-ub-down’ genes.

Recently, another two labs also focused on the complicated interaction of these epigenetic marks in mouse oocytes and early embryos (23,24). Reduction of H2AK119ub1 in oocytes led to H3K27me3 loss at a subset of genes and this accompanying gene-selective H3K27me3 deficiency caused loss of H3K27me3-dependent imprinting in embryos (24). In contrast, H2AK119ub1 depletion in zygotes had no effect on H3K27me3 imprinting maintenance (23). Besides, maternal PRC2 knockout had little effect on global H2AK119ub1 in early embryos but disrupt allelic H2AK119ub1 at H3K27me3 imprinting loci (23). The true director of two modifications would be further studied to clarify. In addition, clear signal for H2AK119ub1 could be found in WT MII oocytes from their CUT&RUN results, which seemed different from ours. We think the key is the unused spike-ins when analyzing their profile. Actually, although CUT&RUN method is sensitive, it is not suitable for direct comparison of signal levels among different samples without spike-ins (59), so we performed H2AK119ub1 profiling using a widely used method based on the conventional ultra-low-input ChIP-seq (i.e. the conventional RPKM normalization) instead of CUT&RUN, and took advantage of the relatively high background to normalize H2AK119ub1 levels among samples (8,20,22,60–62). With this normalization strategy, we could clearly observe a drastic reduction of global H2AK119ub1 levels in MII oocytes, which was also supported by our and other published immunostaining results.

ZGA at the two-cell stage was vital for embryo development. The mRNA profile at the two-cell stage was markedly different from that in oocytes (46–49). Changed histone modifications lead to distinct transcription frequencies of different genes. At the two-cell stage, H2AK119ub1 levels on the promoters of ZGA genes were lower than that of other genes. The decrease in H2AK119ub1 was partly due to its extensive removal by USP16 during meiotic maturation. Genes with decreased H2AK119ub1 levels at the MII and two-cell stages were transcriptionally activated at the two-cell stage. This fact supports our hypothesis that maternal H2AK119ub1 removal after meiosis resumption is vital for ZGA.

Acute H2AK119ub1 depletion at zygote stage caused premature activation of developmental genes during ZGA and subsequent embryonic arrest at the 4-cell stage (23). However, maternal *Usp16*-deleted embryos exhibited severe defects in ZGA and a low developmental potential at the two-cell stage, indicating the significance of USP16-mediated large scale H2AK119ub1 removal from maternal genome during meiosis. USP16 is required for H2AK119ub1 decline during oocyte meiotic maturation because it is the only H2AK119ub1 DUB that is highly expressed in this process. Besides USP16, other DUBs such as USP21 may serve a partially redundant maternal function, because approximately 60% of the *Usp16^♀−^^/^^♂^^+^* and *Usp16^♀−^^/^^♂−^* embryos develop beyond the two-cell stage. Although USP21 expresses at a lower level than USP16, it is biochemically capable of removing ubiquitin from histone H2AK119, as shown in Figure 4I, J. In the absence of USP16, USP21 may still be able to remove a portion of H2K119ub1 from the maternal chromatin, albeit at a slower rate than normal, causing the development of some of the *Usp16*-deleted embryos beyond the two-cell stage. In addition, it is likely that zygotic USP16 continues to play a role in balancing the genomic deposition of H2AK119ub1, because 1) the early zygotic expression of paternal *Usp16* can partially rescue the developmental defects of maternal *Usp16*-deleted embryos; and 2) knockout of paternal *Usp16* strengthens the effect of maternal *Usp16* knockout and leads to complete preimplantation failure in the *Usp16^♀−^^/^^♂−^* embryos. These embryos are different from the previously reported *Usp16* knockout embryos obtained by crossing the *Usp16^+/^^−^* parents, because the latter still contain the maternal deposition of *Usp16* mRNA and proteins from their *Usp16* heterozygous mothers. And *Usp16* knockout caused lethal developmental defects after implantation but before the E7.5 developmental stage. *Usp16^−^^/^^−^* ESCs failed to differentiate due to ubH2A-mediated repression of lineage-specific genes (14). These events well explain why USP16 protein level increased continuously during pre-implantation development. Zygotically expressed USP16 would substitute for maternal USP16 in supporting early embryo development beyond the morula stage and ESC differentiation in after embryo implantation.

Taken together, the accumulation and reduction of maternal H2AK119ub1 in oocytes are both prerequisites for proper embryonic development. The *in vivo* results indicate that the reduction of H2AK119ub1 by an USP16-dependent mechanism during oocyte maturation reduces reprogramming resistance, ensures zygotic genome reprogramming, and thus, provides high developmental competence after fertilization.

## DATA AVAILABILITY

ChIP-seq and RNA-seq data have been deposited in the NCBI Gene Expression Omnibus database under the accession code GSE154412 and GSE169199.

## Supplementary Material

gkac468_Supplemental_File

## References

[1] De La Fuente R. Chromatin modifications in the germinal vesicle (GV) of mammalian oocytes. Dev. Biol. 2006; 292:1–12. 16466710 10.1016/j.ydbio.2006.01.008

[2] Zhou L.Q. , Dean J. Reprogramming the genome to totipotency in mouse embryos. Trends Cell Biol. 2015; 25:82–91. 25448353 10.1016/j.tcb.2014.09.006 PMC4312727

[3] Akiyama T. , Suzuki O., Matsuda J., Aoki F. Dynamic replacement of histone H3 variants reprograms epigenetic marks in early mouse embryos. PLos Genet. 2011; 7:e1002279. 21998593 10.1371/journal.pgen.1002279 PMC3188537

[4] Xu R. , Li C., Liu X., Gao S. Insights into epigenetic patterns in mammalian early embryos. Protein Cell. 2021; 12:7–28. 32671792 10.1007/s13238-020-00757-z PMC7815849

[5] Clarke H.J. , Vieux K.F. Epigenetic inheritance through the female germ-line: the known, the unknown, and the possible. Semin. Cell Dev. Biol. 2015; 43:106–116. 26183189 10.1016/j.semcdb.2015.07.003

[6] Yu C. , Fan X., Sha Q.Q., Wang H.H., Li B.T., Dai X.X., Shen L., Liu J., Wang L., Liu K. et al. CFP1 regulates histone H3K4 trimethylation and developmental potential in mouse oocytes. Cell Rep. 2017; 20:1161–1172. 28768200 10.1016/j.celrep.2017.07.011

[7] Au Yeung W.K. , Brind’Amour J., Hatano Y., Yamagata K., Feil R., Lorincz M.C., Tachibana M., Shinkai Y., Sasaki H. Histone H3K9 methyltransferase G9a in oocytes is essential for preimplantation development but dispensable for CG methylation protection. Cell Rep. 2019; 27:282–293. 30943408 10.1016/j.celrep.2019.03.002

[8] Xu Q. , Xiang Y., Wang Q., Wang L., Brind’Amour J., Bogutz A.B., Zhang Y., Zhang B., Yu G., Xia W. et al. SETD2 regulates the maternal epigenome, genomic imprinting and embryonic development. Nat. Genet. 2019; 51:844–856. 31040401 10.1038/s41588-019-0398-7

[9] Sha Q.Q. , Dai X.X., Jiang J.C., Yu C., Jiang Y., Liu J., Ou X.H., Zhang S.Y., Fan H.Y. CFP1 coordinates histone H3 lysine-4 trimethylation and meiotic cell cycle progression in mouse oocytes. Nat. Commun. 2018; 9:3477. 30154440 10.1038/s41467-018-05930-x PMC6113306

[10] Jullien J. , Vodnala M., Pasque V., Oikawa M., Miyamoto K., Allen G., David S.A., Brochard V., Wang S., Bradshaw C. et al. Gene resistance to transcriptional reprogramming following nuclear transfer is directly mediated by multiple chromatin-repressive pathways. Mol. Cell. 2017; 65:873–884. 28257702 10.1016/j.molcel.2017.01.030 PMC5344684

[11] Matoba S. , Liu Y., Lu F., Iwabuchi K.A., Shen L., Inoue A., Zhang Y. Embryonic development following somatic cell nuclear transfer impeded by persisting histone methylation. Cell. 2014; 159:884–895. 25417163 10.1016/j.cell.2014.09.055 PMC4243038

[12] Matoba S. , Wang H., Jiang L., Lu F., Iwabuchi K.A., Wu X., Inoue K., Yang L., Press W., Lee J.T. et al. Loss of H3K27me3 imprinting in somatic cell nuclear transfer embryos disrupts post-implantation development. Cell Stem Cell. 2018; 23:343–354. 30033120 10.1016/j.stem.2018.06.008 PMC6326833

[13] Liu W. , Liu X., Wang C., Gao Y., Gao R., Kou X., Zhao Y., Li J., Wu Y., Xiu W. et al. Identification of key factors conquering developmental arrest of somatic cell cloned embryos by combining embryo biopsy and single-cell sequencing. Cell Discov. 2016; 2:16010. 27462457 10.1038/celldisc.2016.10 PMC4897595

[14] Yang W. , Lee Y.H., Jones A.E., Woolnough J.L., Zhou D., Dai Q., Wu Q., Giles K.E., Townes T.M., Wang H. The histone H2A deubiquitinase usp16 regulates embryonic stem cell gene expression and lineage commitment. Nat. Commun. 2014; 5:3818. 24784029 10.1038/ncomms4818 PMC4060806

[15] Illingworth R.S. Chromatin folding and nuclear architecture: PRC1 function in 3D. Curr. Opin. Genet. Dev. 2019; 55:82–90. 31323466 10.1016/j.gde.2019.06.006 PMC6859790

[16] Du Z. , Zheng H., Kawamura Y.K., Zhang K., Gassler J., Powell S., Xu Q., Lin Z., Xu K., Zhou Q. et al. Polycomb group proteins regulate chromatin architecture in mouse oocytes and early embryos. Mol. Cell. 2020; 77:825–839. 31837995 10.1016/j.molcel.2019.11.011

[17] Posfai E. , Kunzmann R., Brochard V., Salvaing J., Cabuy E., Roloff T.C., Liu Z., Tardat M., van Lohuizen M., Vidal M. et al. Polycomb function during oogenesis is required for mouse embryonic development. Genes Dev. 2012; 26:920–932. 22499591 10.1101/gad.188094.112 PMC3347790

[18] Eid A. , Torres-Padilla M.E. Characterization of non-canonical polycomb repressive complex 1 subunits during early mouse embryogenesis. Epigenetics. 2016; 11:389–397. 27081692 10.1080/15592294.2016.1172160 PMC4939922

[19] Cao W. , Guo J., Wen X., Miao L., Lin F., Xu G., Ma R., Yin S., Hui Z., Chen T. et al. CXXC finger protein 1 is critical for T-cell intrathymic development through regulating H3K4 trimethylation. Nat. Commun. 2016; 7:11687. 27210293 10.1038/ncomms11687 PMC4879243

[20] Zhang B. , Zheng H., Huang B., Li W., Xiang Y., Peng X., Ming J., Wu X., Zhang Y., Xu Q. et al. Allelic reprogramming of the histone modification H3K4me3 in early mammalian development. Nature. 2016; 537:553–557. 27626382 10.1038/nature19361

[21] Dahl J.A. , Jung I., Aanes H., Greggains G.D., Manaf A., Lerdrup M., Li G., Kuan S., Li B., Lee A.Y. et al. Broad histone H3K4me3 domains in mouse oocytes modulate maternal-to-zygotic transition. Nature. 2016; 537:548–552. 27626377 10.1038/nature19360 PMC6283663

[22] Wang C. , Liu X., Gao Y., Yang L., Li C., Liu W., Chen C., Kou X., Zhao Y., Chen J. et al. Reprogramming of H3K9me3-dependent heterochromatin during mammalian embryo development. Nat. Cell Biol. 2018; 20:620–631. 29686265 10.1038/s41556-018-0093-4

[23] Chen Z. , Djekidel M.N., Zhang Y. Distinct dynamics and functions of H2AK119ub1 and H3K27me3 in mouse preimplantation embryos. Nat. Genet. 2021; 53:551–563. 33821005 10.1038/s41588-021-00821-2 PMC8092361

[24] Mei H. , Kozuka C., Hayashi R., Kumon M., Koseki H., Inoue A. H2AK119ub1 guides maternal inheritance and zygotic deposition of H3K27me3 in mouse embryos. Nature Genetics. 2021; 53:539–550. 33821003 10.1038/s41588-021-00820-3

[25] Nicassio F. , Corrado N., Vissers J.H., Areces L.B., Bergink S., Marteijn J.A., Geverts B., Houtsmuller A.B., Vermeulen W., Di Fiore P.P. et al. Human USP3 is a chromatin modifier required for S phase progression and genome stability. Curr. Biol. 2007; 17:1972–1977. 17980597 10.1016/j.cub.2007.10.034

[26] Joo H.Y. , Jones A., Yang C., Zhai L., Smith A.D., Zhang Z., Chandrasekharan M.B., Sun Z.W., Renfrow M.B., Wang Y. et al. Regulation of histone H2A and H2B deubiquitination and xenopus development by USP12 and USP46. J. Biol. Chem. 2011; 286:7190–7201. 21183687 10.1074/jbc.M110.158311 PMC3044976

[27] Joo H.Y. , Zhai L., Yang C., Nie S., Erdjument-Bromage H., Tempst P., Chang C., Wang H. Regulation of cell cycle progression and gene expression by H2A deubiquitination. Nature. 2007; 449:1068–1072. 17914355 10.1038/nature06256

[28] Zhang X.Y. , Pfeiffer H.K., Thorne A.W., McMahon S.B. USP22, an hSAGA subunit and potential cancer stem cell marker, reverses the polycomb-catalyzed ubiquitylation of histone H2A. Cell Cycle. 2008; 7:1522–1524. 18469533 10.4161/cc.7.11.5962 PMC2709765

[29] Nakagawa T. , Kajitani T., Togo S., Masuko N., Ohdan H., Hishikawa Y., Koji T., Matsuyama T., Ikura T., Muramatsu M. et al. Deubiquitylation of histone H2A activates transcriptional initiation via trans-histone cross-talk with H3K4 di- and trimethylation. Genes Dev. 2008; 22:37–49. 18172164 10.1101/gad.1609708 PMC2151013

[30] Li F. , Han H., Sun Q., Liu K., Lin N., Xu C., Zhao Z., Zhao W. USP28 regulates deubiquitination of histone H2A and cell proliferation. Exp. Cell. Res. 2019; 379:11–18. 30910399 10.1016/j.yexcr.2019.03.026

[31] Pan H. , Jia R., Zhang L., Xu S., Wu Q., Song X., Zhang H., Ge S., Xu X.L., Fan X. BAP1 regulates cell cycle progression through E2F1 target genes and mediates transcriptional silencing via H2A monoubiquitination in uveal melanoma cells. Int. J. Biochem. Cell Biol. 2015; 60:176–184. 25582751 10.1016/j.biocel.2015.01.001

[32] Forster M. , Boora R.K., Petrov J.C., Fodil N., Albanese I., Kim J., Gros P., Nijnik A. A role for the histone H2A deubiquitinase MYSM1 in maintenance of CD8(+) t cells. Immunology. 2017; 151:110–121. 28066899 10.1111/imm.12710 PMC5382346

[33] Gu Y. , Jones A.E., Yang W., Liu S., Dai Q., Liu Y., Swindle C.S., Zhou D., Zhang Z., Ryan T.M. et al. The histone H2A deubiquitinase usp16 regulates hematopoiesis and hematopoietic stem cell function. Proc. Natl. Acad. Sci. U.S.A. 2016; 113:E51–E60. 26699484 10.1073/pnas.1517041113 PMC4711844

[34] Adorno M. , Sikandar S., Mitra S.S., Kuo A., Nicolis Di Robilant B., Haro-Acosta V., Ouadah Y., Quarta M., Rodriguez J., Qian D. et al. Usp16 contributes to somatic stem-cell defects in down's syndrome. Nature. 2013; 501:380–384. 24025767 10.1038/nature12530 PMC3816928

[35] Xu J.C. , Dawson V.L., Dawson T.M. Usp16: key controller of stem cells in down syndrome. EMBO J. 2013; 32:2788–2789. 24076652 10.1038/emboj.2013.220 PMC3817468

[36] Montellese C. , van den Heuvel J., Ashiono C., Dorner K., Melnik A., Jonas S., Zemp I., Picotti P., Gillet L.C., Kutay U. USP16 counteracts mono-ubiquitination of RPS27a and promotes maturation of the 40S ribosomal subunit. Elife. 2020; 9:e54435. 32129764 10.7554/eLife.54435 PMC7065907

[37] Zhang Y. , Liu R.B., Cao Q., Fan K.Q., Huang L.J., Yu J.S., Gao Z.J., Huang T., Zhong J.Y., Mao X.T. et al. USP16-mediated deubiquitination of calcineurin a controls peripheral T cell maintenance. J. Clin. Invest. 2019; 129:2856–2871. 31135381 10.1172/JCI123801 PMC6597231

[38] Lan Z.J. , Xu X., Cooney A.J. Differential oocyte-specific expression of cre recombinase activity in GDF-9-iCre, Zp3cre, and MSX2CRE transgenic mice. Biol. Reprod. 2004; 71:1469–1474. 15215191 10.1095/biolreprod.104.031757

[39] Zhu Y. , Yu J., Rong Y., Wu Y.-W., Li Y., Zccchang L., Pan Y., Fan H.-Y., Shen L.J.S.B. Genomewide decoupling of H2AK119ub1 and H3K27me3 in early mouse development. 2021; 66:2489–2497. 10.1016/j.scib.2021.06.010 36654208

[40] Brind’Amour J. , Liu S., Hudson M., Chen C., Karimi M.M., Lorincz M.C. An ultra-low-input native chip-seq protocol for genome-wide profiling of rare cell populations. Nat. Commun. 2015; 6:6033. 25607992 10.1038/ncomms7033

[41] Tan J.H. , Wang H.L., Sun X.S., Liu Y., Sui H.S., Zhang J. Chromatin configurations in the germinal vesicle of mammalian oocytes. Mol. Hum. Reprod. 2009; 15:1–9. 19019837 10.1093/molehr/gan069

[42] Yan L. , Yang M., Guo H., Yang L., Wu J., Li R., Liu P., Lian Y., Zheng X., Yan J. et al. Single-cell RNA-Seq profiling of human preimplantation embryos and embryonic stem cells. Nat. Struct. Mol. Biol. 2013; 20:1131–1139. 23934149 10.1038/nsmb.2660

[43] Zhuo X. , Guo X., Zhang X., Jing G., Wang Y., Chen Q., Jiang Q., Liu J., Zhang C. Usp16 regulates kinetochore localization of plk1 to promote proper chromosome alignment in mitosis. J. Cell Biol. 2015; 210:727–735. 26323689 10.1083/jcb.201502044 PMC4555819

[44] Sen Nkwe N. , Daou S., Uriarte M., Gagnon J., Iannantuono N.V., Barbour H., Yu H., Masclef L., Fernandez E., Zamorano Cuervo N. et al. A potent nuclear export mechanism imposes USP16 cytoplasmic localization during interphase. J. Cell Sci. 2020; 133:jcs239236. 32005696 10.1242/jcs.239236

[45] Howe F.S. , Fischl H., Murray S.C., Mellor J. Is H3K4me3 instructive for transcription activation?. Bioessays. 2017; 39:1–12. 10.1002/bies.201600095 28004446

[46] Tadros W. , Lipshitz H.D. The maternal-to-zygotic transition: a play in two acts. Development. 2009; 136:3033–3042. 19700615 10.1242/dev.033183

[47] Vastenhouw N.L. , Cao W.X., Lipshitz H.D. The maternal-to-zygotic transition revisited. Development. 2019; 146:dev161471. 31189646 10.1242/dev.161471

[48] Yu C. , Ji S.Y., Sha Q.Q., Dang Y., Zhou J.J., Zhang Y.L., Liu Y., Wang Z.W., Hu B., Sun Q.Y. et al. BTG4 is a meiotic cell cycle-coupled maternal-zygotic-transition licensing factor in oocytes. Nat. Struct. Mol. Biol. 2016; 23:387–394. 27065194 10.1038/nsmb.3204

[49] Abe K. , Yamamoto R., Franke V., Cao M., Suzuki Y., Suzuki M.G., Vlahovicek K., Svoboda P., Schultz R.M., Aoki F. The first murine zygotic transcription is promiscuous and uncoupled from splicing and 3' processing. EMBO J. 2015; 34:1523–1537. 25896510 10.15252/embj.201490648 PMC4474528

[50] Macfarlan T.S. , Gifford W.D., Driscoll S., Lettieri K., Rowe H.M., Bonanomi D., Firth A., Singer O., Trono D., Pfaff S.L. Embryonic stem cell potency fluctuates with endogenous retrovirus activity. Nature. 2012; 487:57–63. 22722858 10.1038/nature11244 PMC3395470

[51] Zhang W. , Chen F., Chen R., Xie D., Yang J., Zhao X., Guo R., Zhang Y., Shen Y., Goke J. et al. Zscan4c activates endogenous retrovirus MERVL and cleavage embryo genes. Nucleic Acids Res. 2019; 47:8485–8501. 31304534 10.1093/nar/gkz594 PMC7145578

[52] Ruberto A.A. , Grechez-Cassiau A., Guerin S., Martin L., Revel J.S., Mehiri M., Subramaniam M., Delaunay F., Teboul M. KLF10 integrates circadian timing and sugar signaling to coordinate hepatic metabolism. *eLife*. 2021; 10:e65574. 10.7554/eLife.65574 PMC8410083 34402428

[53] Desai D. , Pethe P. Polycomb repressive complex 1: regulators of neurogenesis from embryonic to adult stage. J. Cell. Physiol. 2020; 235:4031–4045. 31608994 10.1002/jcp.29299

[54] Marsh D.J. , Dickson K.A. Writing histone monoubiquitination in human malignancy-the role of RING finger E3 ubiquitin ligases. Genes (Basel). 2019; 10:67. 10.3390/genes10010067 PMC6356280 30669413

[55] Vissers J.H. , Nicassio F., van Lohuizen M., Di Fiore P.P., Citterio E. The many faces of ubiquitinated histone H2A: insights from the DUBs. Cell Div. 2008; 3:8. 18430235 10.1186/1747-1028-3-8 PMC2373781

[56] Simon J.A. , Kingston R.E. Mechanisms of polycomb gene silencing: knowns and unknowns. Nat. Rev. Mol. Cell Biol. 2009; 10:697–708. 19738629 10.1038/nrm2763

[57] Vastenhouw N.L. , Schier A.F. Bivalent histone modifications in early embryogenesis. Curr. Opin. Cell Biol. 2012; 24:374–386. 22513113 10.1016/j.ceb.2012.03.009 PMC3372573

[58] Sachs M. , Onodera C., Blaschke K., Ebata K.T., Song J.S., Ramalho-Santos M. Bivalent chromatin marks developmental regulatory genes in the mouse embryonic germline in vivo. Cell Rep. 2013; 3:1777–1784. 23727241 10.1016/j.celrep.2013.04.032 PMC3700580

[59] Meers M.P. , Bryson T.D., Henikoff J.G., Henikoff S. Improved CUT&RUN chromatin profiling tools. Elife. 2019; 8:e46314. 31232687 10.7554/eLife.46314 PMC6598765

[60] Hanna C.W. , Taudt A., Huang J., Gahurova L., Kranz A., Andrews S., Dean W., Stewart A.F., Colome-Tatche M., Kelsey G. MLL2 conveys transcription-independent H3K4 trimethylation in oocytes. Nat. Struct. Mol. Biol. 2018; 25:73–82. 29323282 10.1038/s41594-017-0013-5

[61] Liu Z. , Tardat M., Gill M.E., Royo H., Thierry R., Ozonov E.A., Peters A.H. SUMOylated PRC1 controls histone H3.3 deposition and genome integrity of embryonic heterochromatin. EMBO J. 2020; 39:e103697. 32395866 10.15252/embj.2019103697 PMC7327501

[62] Zheng H. , Huang B., Zhang B., Xiang Y., Du Z., Xu Q., Li Y., Wang Q., Ma J., Peng X. et al. Resetting epigenetic memory by reprogramming of histone modifications in mammals. Mol. Cell. 2016; 63:1066–1079. 27635762 10.1016/j.molcel.2016.08.032

