# Supplementary material for: USP16-mediated histone H2A lysine-119 deubiquitination during oocyte maturation is a prerequisite for zygotic genome activation
Source: Nucleic Acids Res. 2022 May 30;50(10):5599–616. doi: 10.1093/nar/gkac468 (PMC9178006; doi:10.1093/nar/gkac468)
Supplement: gkac468_Supplemental_File [file gkac468_supplemental_file.pdf]

Supplementary Information
Supplementary Figures

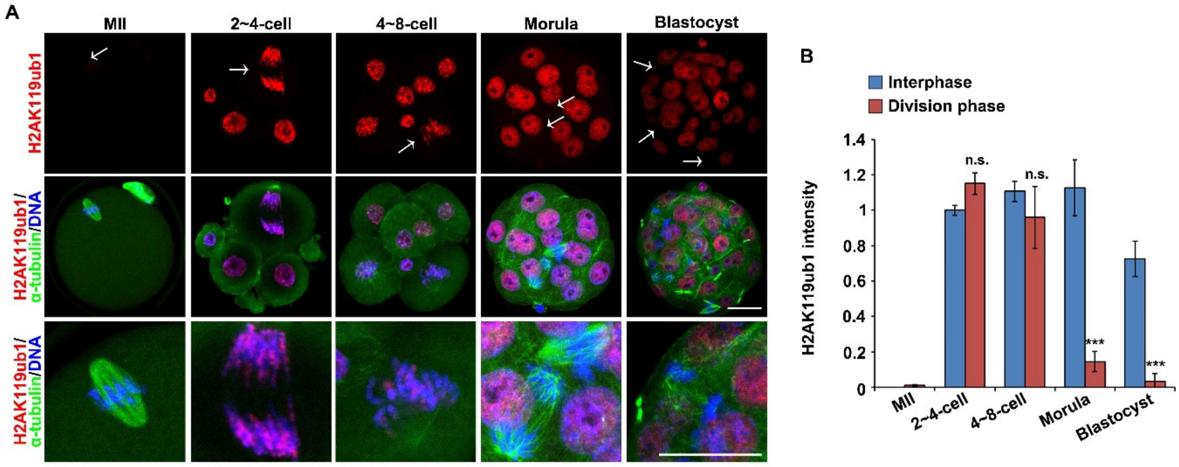

Figure S1. H2AK119ub1 decline was observed in meiosis but not at every division phase of embryonic development.

A: H2AK119ub1 immunofluorescence results in dividing mouse oocytes and preimplantation embryos. Arrows indicated the oocyte or blastomeres at the division phase. The third line was the zoom pictures of chromosomes. Scale bar, 20 μm. **B:** Quantification of H2AK119ub1 signal intensity of the oocyte or blastomeres at interphase and division phase from **(A)**. The numbers of analyzed oocytes and embryos at each stage were more than eight. Error bars, S.E.M. ***P < 0.001 using two-tailed Student's t-tests. n.s.: non-significant.

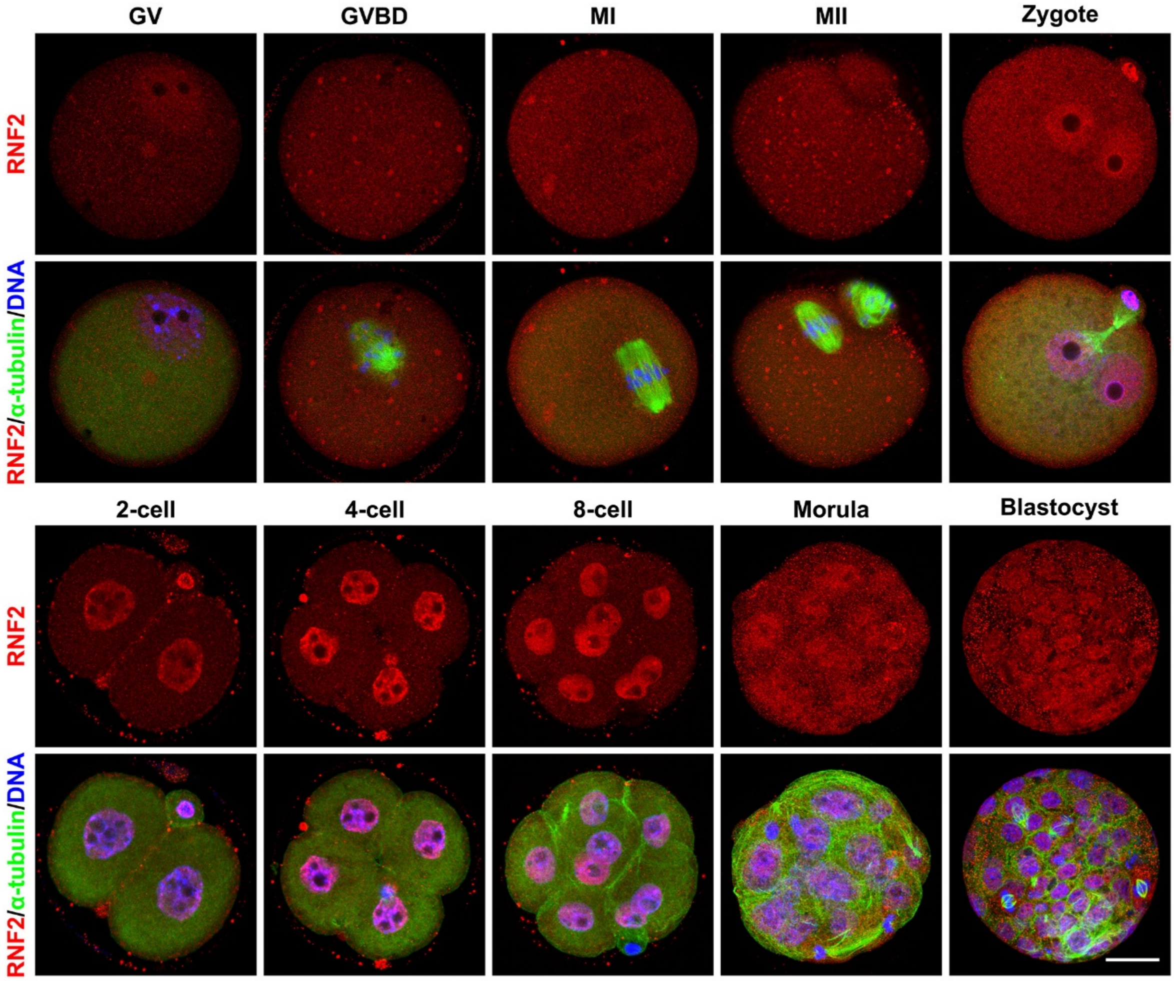

Figure S2. RNF2 immunofluorescence results in mouse oocytes and preimplantation embryos. The numbers of analyzed oocytes or embryos at each stage were more than eight. Scale bar, 20 μm.

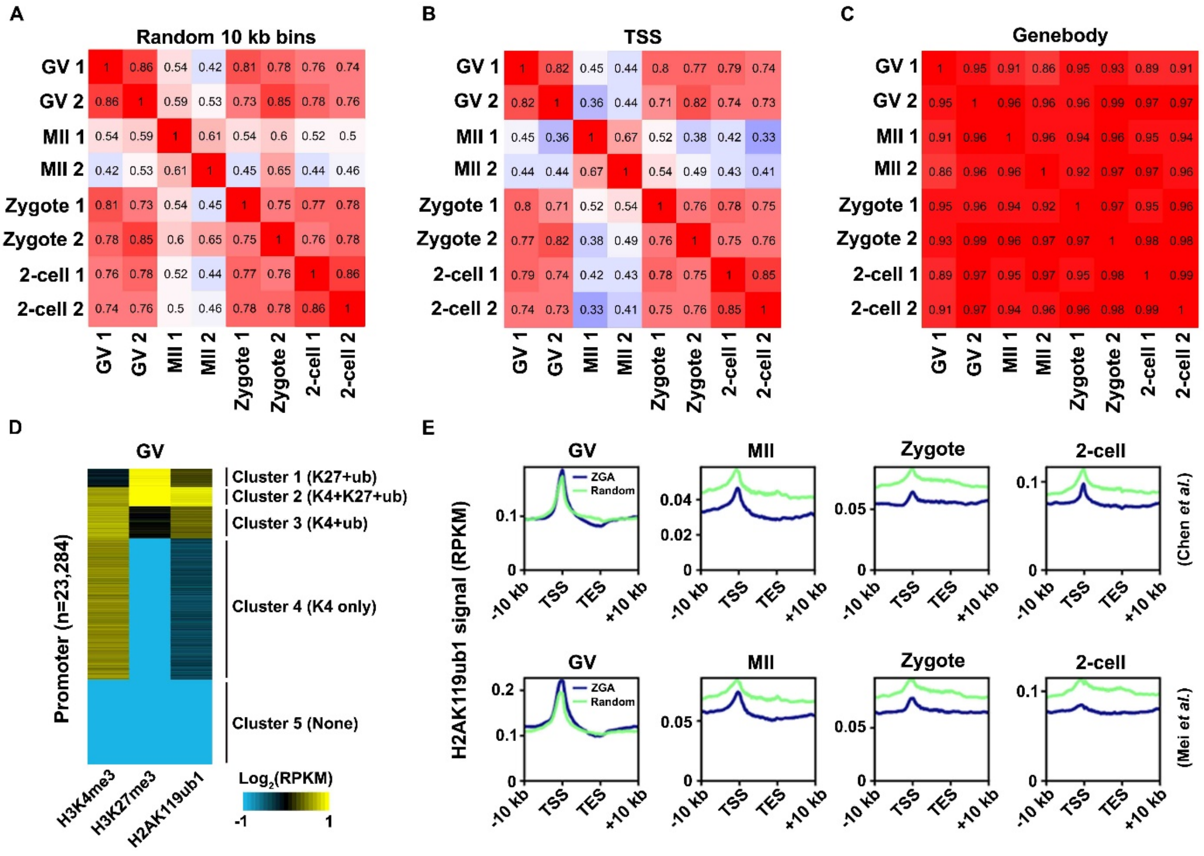

Figure S3. Analysis of H2AK119ub1 ChIP-seq data in oocytes and embryos.
A-C: Heat map showing correlation of H2AK119ub1 ChIP-seq replicates at each 10 kb bins across the genome **(A)**, TSSs **(B)** and gene bodies **(C)** of the genes. **D:** Heat map showing histone modification enrichment at individual promoters in GV oocytes. Promoters are clustered into five groups using k-means clustering and further defined manually based on H3K4me3, H3K27me3 and H2AK119ub1 signals. **E:** Metaplot showing H2AK119ub1 enrichment of ZGA genes and random genes in GV and MII oocytes, zygotes, and 2-cell stage embryos (data obtained from Chen *et al.* and Mei *et al.*).

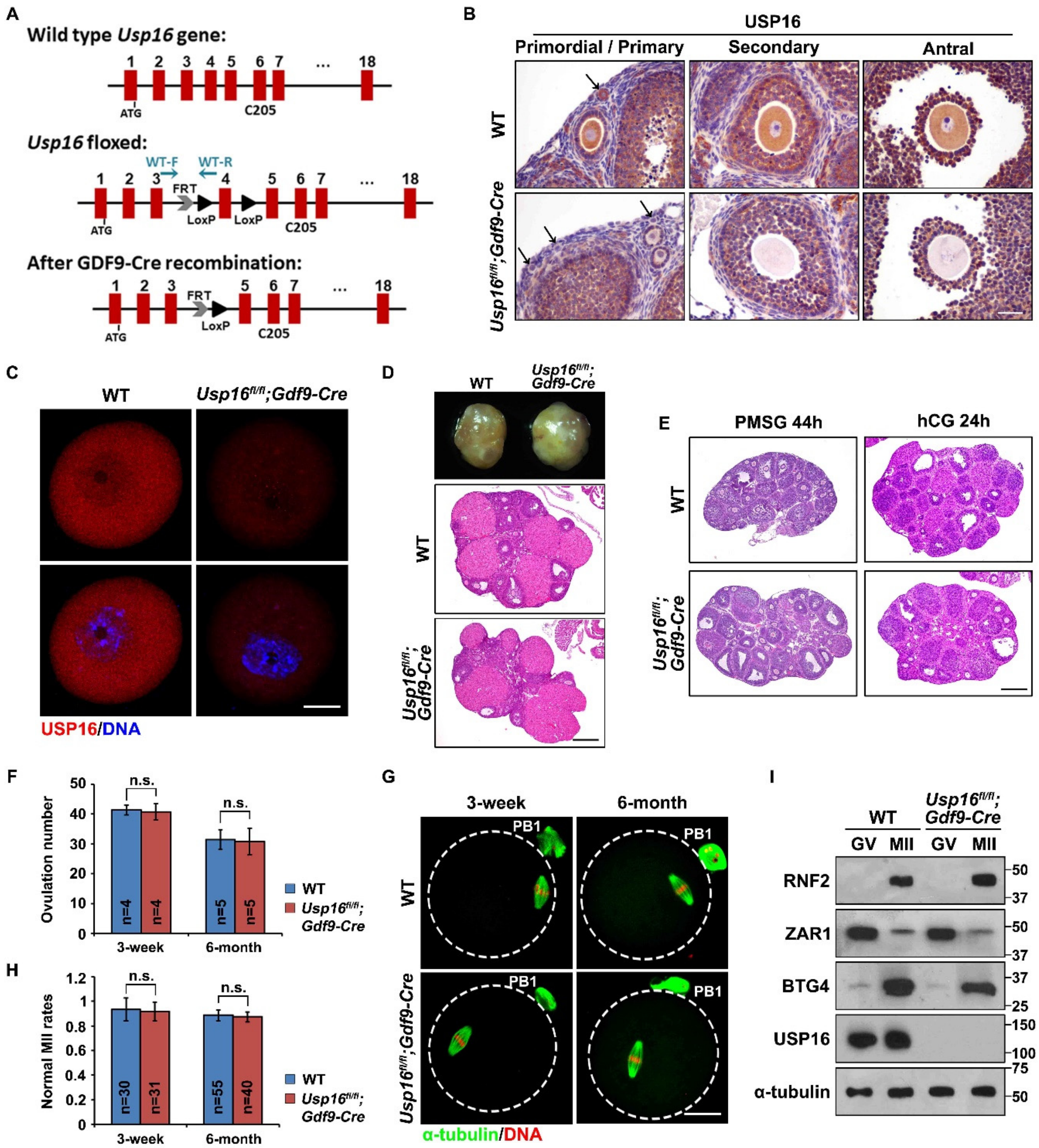

Figure S4. Oocyte-specific knockout of *Usp16* and phenotype analyses.

A: Schematic representation of the strategy used to generate oocyte-specific *Usp16* null alleles. *Gdf9-Cre*-mediated recombination resulted in an in-frame deletion. PCR primers used for genotyping have been indicated with blue arrows. **B:** IHC results showing USP16 protein levels in the ovaries of WT and *Usp16$^{fl/fl}$;Gdf9-Cre* mice. The stages of follicles have been indicated. Arrows indicated oocytes within primordial follicles. Scale bar, 50 µm. For each genotype, at least three females were used. **C:** Immunofluorescence results showing USP16 protein levels in the oocytes from WT and *Usp16$^{fl/fl}$;Gdf9-Cre* mice. **D:** Images and H&E staining results of ovaries from 8-month-old WT and *Usp16$^{fl/fl}$;Gdf9-Cre* female mice. Scale bar, 500 µm. **E:** H&E staining results of ovarian histology in 3-week-old WT and *Usp16$^{fl/fl}$;Gdf9-Cre* mice at 44 h after PMSG injection (top) and at 24 h after hCG injection (bottom). Scale bar, 500 µm. **F:** The average numbers of ovulated oocytes in WT and *Usp16$^{fl/fl}$;Gdf9-Cre* females at 16 h after hCG

injection, as determined using a superovulation assay. The numbers of analyzed mice have been indicated (n). Error bars, S.E.M. n.s.: non-significant. **G:** Immunofluorescence staining results showing spindles of oocytes collected from oviducts of WT and *Usp16*$^{fl/fl}$*;Gdf9-Cre* females at 16 h after hCG injection. Scale bar, 20 μm. **H:** Rates of normal spindle assembly in MII oocytes. The numbers of analyzed oocytes have been indicated (n). Error bars, S.E.M. n.s.: non-significant. **I:** Western blot results showing maternal protein expression in oocytes of WT and *Usp16*$^{fl/fl}$*;Gdf9-Cre* females. Total proteins from 100 oocytes were loaded in each lane. α-tubulin was blotted as a loading control.

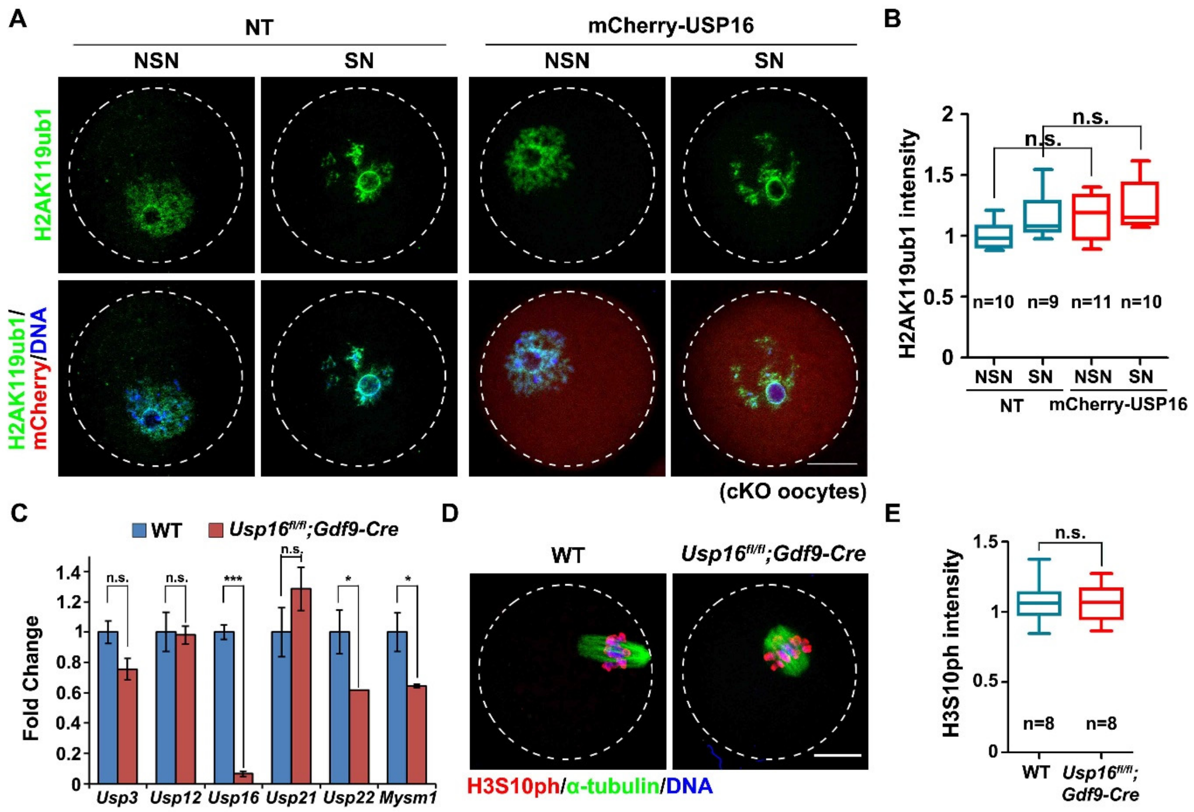

Figure S5. The effect of deleting maternal USP16 on other DUBs and histone modifications.

A: Levels of H2AK119ub1 in *Usp16*-null oocytes and *Usp16*-null oocytes microinjected with mRNAs encoding mCherry-USP16. Scale bar, 20 μm. **B:** Quantification of H2AK119ub1 signal intensity of **(A)**. The numbers of analyzed oocytes at each stage were indicated. Error bars, S.E.M. n.s.: non-significant. **C:** RT-qPCR results showing mRNA levels of indicated DUBs in GV oocytes of WT and *Usp16$^{fl/fl}$;Gdf9-Cre* females. n = 3 biological replicates. Error bars, SEM. **P* < 0.05, ****P* < 0.001 by two-tailed Student's *t*-test. n.s.: non-significant. **D:** Levels of H3S10ph in MI oocytes of WT and *Usp16$^{fl/fl}$;Gdf9-Cre* females. Scale bar, 20 μm. **E:** Quantification of H3S10ph signal intensity in MI oocytes of WT and *Usp16$^{fl/fl}$;Gdf9-Cre* females. The numbers of analyzed oocytes have been indicated (n). Error bars, SEM. n.s.: non-significant.

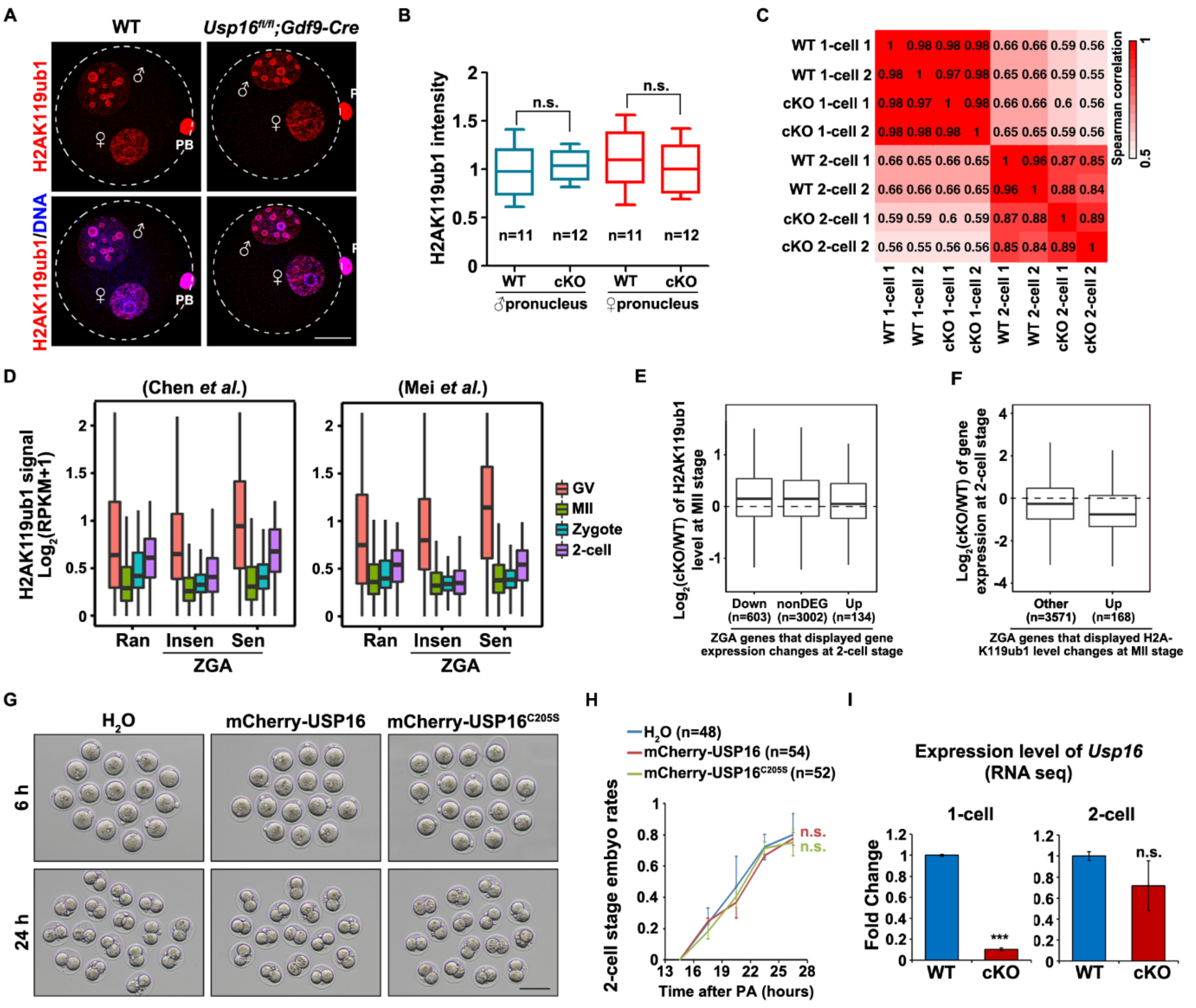

Figure S6. USP16 deletion in oocytes caused ZGA failure in the 2-cell embryo.

A: H2AK119ub1 levels in zygotes from WT and *Usp16$^{fl/fl}$;Gdf9-Cre* females. Scale bar, 20 μm. **B:** Quantification of H2AK119ub1 signal intensity of **(A)**. The numbers of analyzed oocytes at each stage were indicated. Error bars, S.E.M. n.s.: non-significant. **C:** Heat map showing spearman correlation coefficients among WT and maternal *Usp16* knockout embryos at different stages. **D:** Boxplot showing H2AK119ub1 enrichment at USP16-insensitive and -sensitive ZGA genes at indicated stages by the public CUT&RUN data. **E:** Boxplot showing H2AK119ub1 level changes of indicated gene sets at the MII stage after *Usp16* deletion. **F:** Boxplot showing gene expression changes of indicated gene sets at the 2-cell stage after *Usp16* deletion. **G:** Representative images of parthenogenetic embryos microinjected with H_2O or mRNAs encoding USP16 (WT or USP16^{C205S}). Scale bar, 100 μm. **H:** Developmental rates of parthenogenetic embryos microinjected with H_2O or mRNAs encoding USP16 (WT or USP16^{C205S}). The numbers of analyzed embryos have been indicated (n). Error bars, S.E.M. n.s.: non-significant. **I:** Bar plot showing the expression level of *Usp16* from RNA-seq data. Error bars, S.E.M. ***$P < 0.001$ by two-tailed Student's *t*-test. n.s.: non-significant.

Supplementary Tables

Supplementary Table 1. Antibody information.

Protein name	Manufacture (catalogue number)	Applications (working dilution)
USP16	Abcam (ab236628)	WB (1:500); IF (1:50); IHC (1:50)
RNF2	Proteintech (16031-1-AP)	WB (1:1000); IF (1:200); IHC (1:200)
ZAR1	Made by Abcam company	WB (1:3000)
BTG4	Abcam (ab206914)	WB (1:500)
FITC-α-tubulin	Sigma (F2168)	IF (1:200)
α-tubulin (11H10)	Cell Signaling (2125S)	WB (1:500)
GAPDH	Proteintech (60004-1-Ig)	WB (1:5000)
CREST	Fitzgerald Industries International (70R-21494)	IF (1:100)
H2AK119ub1	Cell Signaling (8240)	IF (1:400); IHC (1:400); ChIP (1-1.5μg/sample)
H3S10ph	Cell Signaling (9701S)	IF (1:100)
PolIISer2P	Abcam (ab5095)	IF (1:40000)
CDX2	BioGenex (AM392-5M)	Ready to use, IF
Nanog	Cell Signaling (8822S)	IF (1:100)

Supplementary Table 2. Primer sequences.

Primer name	Target Gene	Application	Sequences (5′-3′)
Usp16 WT-F	*Usp16*	Genotyping of WT /Flox allele	5′- GCAGAGGGTCTGACGGATGTG -3′
Usp16 WT-R			5′- CAAAATACACTCTACAGAGTCTGGAGG -3′
Usp16-F		Real-time PCR /Absolute RT-qPCR	5′- CTATGAGGTCAGGGCATTACACTGC -3′
Usp16-R			5′- GCTTGCACATGTGTATCGCTGATG -3′
Zfp352-F	*Zfp352*	Real-time PCR	5′- AAGTCCCACATCTGAAGAAACAC -3′
Zfp352-R			5′- GGGTATGAGGATTCACCCACA -3′
Zscan4c-F	*Zscan4c*	Real-time PCR	5′- GCCTTATGTCTGTTCCCTATGT -3′
Zscan4c-R			5′- CAGTCTCTGCTGAGGATGTTAG -3′
Zscan4f-F	*Zscan4f*	Real-time PCR	5′- CTGATGAGTGCTTGAAGCCTC -3′
Zscan4f -R			5′- TCCACTACAGCTTTCACCAAC -3′
Klf10-F	*Klf10*	Real-time PCR	5′- ATGCTCAACTTCGGCGCTT -3′
Klf10-R			5′- CGCTTCCACCGCTTCAAAG -3′

Cdk16-F	*Cdk16*	Real-time PCR	5′- AGATAAGACCAATGGTGTCCCT -3′
Cdk16-R			5′- CACTCTCCCCATCAGATCCCA -3′
Phlda2-F	*Phlda2*	Real-time PCR	5′- CTCCGACGAGATCCTTTGCG -3′
Phlda2-R			5′- ACACGTACTTAGAGGTGTGCTC -3′
Ankrd22-L-F	*Ankrd22*	Real-time PCR	5′- CAGCCTACCAGAACGACTTGG -3′
Ankrd22-R			5′- GATGAGGGGAGTATCGCCATT -3′
Hspa2-F	*Hspa2*	Real-time PCR	5′- GCGTGGGGGTATTCCAACAT -3′
Hspa2-R			5′- TGAGACGCTCGGTGTCAGT -3′
Usp3-F	*Usp3*	Real-time PCR	5′- TCAGCCAAGTTCCCCAACG -3′
Usp3-R			5′- GCCATTCACATACCTTCCACAGT -3′
Usp12-F	*Usp12*	Real-time PCR	5′- ACAGTCTCCAAATTCGCCTCC -3′
Usp12-R			5′- ACTGAGTTGCAGTAGCAGGTATT -3′
Usp21-F	*Usp21*	Real-time PCR /Absolute RT-qPCR	5′- GGACTTTGCCAGCGACAAAGC -3′
Usp21-R			5′- GCCACCTGATTTTCACTGACAGG -3′
Usp22-F	*Usp22*	Real-time PCR	5′- CCAAACTTCGGCGGAAGATCAC -3′
Usp22-R			5′-CTTGAACCACTGGTCTTTGTGCTGC -3′
Mysm1-F	*Mysm1*	Real-time PCR	5′- TGCACAGCCGGGAAATGAT -3′
Mysm1-R			5′- ATGGTGCTATCCAGAGTCCAA -3′
Gapdh-F	*Gapdh*	Real-time PCR	5′- ACACTGAGGACCAGGTTGTCTC -3′
Gapdh-R			5′- TACTCCTTGGAGGCCATGTAG -3′
Gfp-F	*Gfp*	Quantitative RT-qPCR	5′- CGCTACCCCGACCACATGAA -3′
Gfp-R			5′- CTTCAGCTCGATGCGGTTCA -3′
